# Temperature-Dependent Modeling and Spatial Predictions for Identifying Geographical Areas in Brazil Suitable for the Use of *Cordyceps javanica* in Whitefly Control

**DOI:** 10.3390/jof11020125

**Published:** 2025-02-08

**Authors:** Heloiza A. Boaventura, Lidiane A. Queirós, José Francisco A. Silva, Tarryn A. Goble, Kelly Pazolini, Allan F. Marciano, Eliane D. Quintela

**Affiliations:** 1 Escola de Agronomia, Universidade Federal de Goiás, Goiânia 74690-900, Brazil; lidianekeiroz@gmail.com; 2 Embrapa Arroz e Feijão, Santo Antônio de Goiás 75375-000, Brazil; jose.arruda-silva@embrapa.br; 3 Lallemand Plant Care, Patos de Minas 38706-420, Brazil; tgoble@lallemand.com (T.A.G.); kpazolini@lallemand.com (K.P.); amarciano@lallemand.com (A.F.M.)

**Keywords:** *Bemisia tabaci*, entomopathogenic fungi, mathematical models, control, IPM

## Abstract

Lalguard C99 WP, based on the *Cordyceps javanica* BRM 27666 strain, is registered in Brazil for whitefly control. Spatial prediction is crucial for optimizing its field use and efficacy. In this study, the optimal temperature for mycelial growth and conidial production of *C. javanica is* 25–30 °C, with no growth at 33–35 °C. The highest nymphal mortality occurred at 25 and 30 °C, showing lower LT_50_ values at 30 °C. Mycelial growth was similar at 15, 20, 25, 30, and 35 °C when the fungus was exposed for 6 h and then transferred to a 27.4 °C environment; however, growth was slower at 35 °C with daily 6 h exposure alternating over 18 h at room temperature (mean of 28.5 °C). When the second instar whitefly nymphs were exposed for 6 h or 6 h daily at 15, 20, 25, 30, and 35 °C, followed by 7 days at fluctuating temperatures (mean of 28.4–30.2 °C), nymphal mortality was similar across temperatures. Although other abiotic factors (solar radiation, humidity, rainfall, etc.) must be considered for fungal efficacy, spatial predictions based on fluctuating temperatures indicated that *C. javanica* is suitable for use throughout Brazil, though its performance varied at constant temperatures in different locations.

## 1. Introduction

*Cordyceps javanica* (Frieder. & Bally) (Hypocreales: Cordycipitaceae) is an entomopathogenic fungus capable of infecting a wide range of agricultural pests [1]. This species is the most prevalent *Cordyceps* species in Brazil, originally isolated from both soil and various insect orders, and is predominant within populations of the whitefly, *Bemisia tabaci* (Hemiptera: Aleyrodidae) [2,3]. Many strains previously referred to as *C. fumosorosea* were shown to be *C. javanica*, such as the strains ESALQ 1296 and PFR-97, active ingredients of commercial mycoinsecticides registered in Brazil and USA, respectively, used for whitefly control [3].

In Central Brazil, several *C. javanica* strains were isolated from whiteflies under epizootic conditions [2]. Since 2012, the Embrapa Arroz e Feijão research team has conducted studies in the laboratory, screenhouse, and field to select the best *C. javanica* strain for a bioproduct development based on mass production characteristics, tolerance to detrimental environmental factors (UV radiation, high temperatures, oxidative, and osmotic stress), and virulence to all stages of *B. tabaci* [4,5,6]. Then, after seven years of a collaborative research project between Embrapa and Lallemand Plant Care (Patos de Minas, MG, Brazil), a product named Lalguard C99 based on aerial conidia of the *C. javanica* strain BRM 27666 formulated as a wettable powder was registered in August 2022 in Brazil. This was the first biopesticide based on this fungus species for the control of *B. tabaci* in Brazil [7], and in 2023, it was also registered in Paraguay.

The whitefly, *B. tabaci*, is an economically significant pest in Brazil and damages more than 40 agricultural crops [2,8]. Prior to the introduction of biotype B throughout the American Tropics, *B. tabaci* was an important pest and virus vector in bean and tomato crops only [2,9]. Following its introduction in 1989, the spread and increase in population size of biotype B was facilitated by the agricultural farming system of Brazil (three growing seasons), combined with favorable climatic conditions [2,10]. This pest established itself and continues to persist in cotton, dry bean, soybean, tomato, and several other crops and wild plant species.

The increased area planted with soybean is credited as one of the main factors driving the development of *B. tabaci* in Brazil, often reaching extremely high population levels and resulting in enormous ‘clouds’ of whiteflies [2,10,11]. In 30 years, the increase in soybean production area jumped by 306%, from 10.7 million hectares to 45.2 million hectares in the 2022/23 season [12]. In soybeans, damage may be caused by direct feeding and indirectly by deposition of honeydew, on which sooty mold fungi can grow and impede photosynthesis, and by transmission of the stem necrosis CpMMV (Cowpea mild mottle virus) [13,14,15].

In general, soybeans are cultivated from October to March in Brazil when high infestation levels of *B. tabaci* can be observed, particularly in the ‘veranico’ period, characterized by cycles of hot and dry days (lasting for 2–4 weeks) in the central–west (Mato Grosso, Mato Grosso do Sul, Goiás, and Federal District), north (Tocantins), and northeast (Maranhão, Piauí, and Bahia) [11,16,17]. In these regions, soybean production can suffer from the high incidence of whiteflies, intensified by high temperatures that reduce their life cycle and increase the number of generations [18].

Fungal efficacy is intrinsically mediated by abiotic factors, especially humidity, temperature, rainfall, and solar radiation [19,20,21,22,23]. For humidity, an increasing number of studies indicated that sufficient moisture exists within the microhabitat of many insect hosts or within the microenvironment of the host’s body surface to support infection independent of ambient moisture conditions [24,25,26,27,28,29]. A study by Wraight et al. [30] demonstrated that a moisture-saturated environment was not required for *C. fumosorosea* infection of *B. tabaci* nymphs on excised hibiscus leaves, and it could infect third instars incubated at 25–30% RH. Boaventura et al. [6] also showed the ability of *C. javanica* to exploit the moist conditions of the leaf or insect boundary layer for germination and host infection, and the low temperature seemed to be more detrimental for *Cordyceps* virulence to *Bemisia* nymphs than RH. Furthermore, in the field, the soybean canopy microclimate is also a factor that favors fungal infection in the host (dew formation that creates a humid environment near the vicinity of the leaf) [31].

Furthermore, temperature influences all fungal development stages, including viability, morphogenesis, germination, host interaction, virulence, and conidiogenesis [32,33]. *Cordyceps* spp. are known to be mesophilic since they grow over a range of 8 to 32 °C, with thermal optima ranging from 20 to 30 °C and limits at 35 °C. Their thermal tolerance is related to their history, including their geo-climatic origin [34,35,36,37]. Boaventura et al. [6] observed that temperatures ≥ 35 °C for 4 to 6 h daily did not affect the efficacy of the three strains of *C. javanica*, including the strain BRM 27666, against nymphs.

In Brazil, climate varies considerably depending on factors such as latitude, sea conditions, and air masses. Thus, various climate types can be identified in Brazil: equatorial (hot and humid, with year-round rainfall and temperatures between 26 and 28 °C, reaching 35 °C in summer); semi-arid (hot and dry, with low rainfall and temperatures above 25 °C, often exceeding 28 °C); tropical (dry winters and rainy summers, with temperatures ranging from 18 to 27 °C, occasionally exceeding 30 °C in the hottest months); high-altitude tropical (distinct winter and summer seasons, with temperatures ranging from 15 to 22 °C and frost occurring in some areas); tropical Atlantic (encompassing the coastal region, with temperatures ranging from mild to hot, between 18 and 26 °C); and subtropical (mild climate, with temperatures ranging from 18 °C to below 0 °C in winter, with high temperatures in summer) [38]. Planting areas in Brazil are predominantly located in a tropical climate.

To further elucidate how this fungus, specifically the strain BRM 27666, will perform in different temperature environments, this study was conducted to explore the environmental factors associated with the virulence of *C. javanica* to the whitefly, *B. tabaci*. Thus, the objectives of this work were to develop temperature-dependent modeling and spatial prediction areas suited for the use of *C. javanica* BRM 27666 for whitefly control in Brazil, according to studies of mycelial growth, conidial production, and virulence under different temperature regimes.

## 2. Materials and Methods

This study resulted from a collaboration between Embrapa and Lallemand Plant Care (Patos de Minas, Minas Gerais, Brazil) under a collaborative research and development agreement. The experiments were conducted in the Insect Pathology Laboratory and Screenhouse at the Brazilian Agricultural Research Corporation (Embrapa Rice and Beans) located in Santo Antônio de Goiás, Goiás state (Central Brazil) (16°30′24.57″ S, 49°17′06.53″ W).

### 2.1. Insect Colony

The whitefly, *B. tabaci*, used in all experiments was identified as biotype B by molecular gene sequence markers from mtDNA cytochrome oxidase I (mtCOI) [2]. The whiteflies originated from a colony reared on common bean plants (*Phaseolus vulgaris* L., cv. Pérola) maintained under screenhouse with no environmental control at the Embrapa Rice and Beans Research Station.

### 2.2. Fungal Strain and Preparations

The *C. javanica* strain BRM 27666 was originally isolated from infected *B. tabaci* adults collected from soybeans in Porangatu, Goiás state, Central Brazil (13°27′ S, 49°10′ W, 600 m altitude). The region has a tropical savanna climate (Aw, megathermal) per Köppen classification, with two distinct seasons: a dry season (May to September) and a rainy season (October to April). The average annual temperature is 26 °C, reaching up to 39 °C in September and October [39]. This strain was preserved in liquid nitrogen and deposited at the Invertebrate Fungal Collection at Embrapa Genetic Resources and Biotechnology (Brasília, DF, Brazil) and in the Invertebrate Fungal Collection at Embrapa Rice and Beans. The *C. javanica* was identified using multi-loci-based phylogenetic methods, according to Lopes et al. [3].

For the mycelial growth and conidiogenesis experiments, the fungus was cultured on potato dextrose agar (PDA) in 9 cm Petri plates in the dark at 26 ± 1 °C for 7–10 days. After this period, the conidia were suspended in 10 mL of sterile aqueous solution of 0.01% (*v*/*v*) Tween 80 in 50 mL plastic centrifuge tubes. The suspension was vigorously agitated on a vortex mixer for 1 min and filtered through two layers of 30 μm pore-sized nylon cheesecloth. The filtered suspension (10 mL) was vortexed again for 1 min before application, and conidial concentrations were enumerated by a hemocytometer (Brightline Improved Neubauer, New Optik^®^, Brazil) at 400× magnification.

For the virulence experiments, the commercial product Lalguard C99 containing conidia of the strain BRM 27666 formulated as a wettable powder was provided by Lallemand Plant Care (Patos de Minas, MG, Brazil). For all experiments, conidial germination exceeded 90% on PDA after 16 h in darkness at 26 ± 1 °C. Only conidia with germ tubes greater than the conidial diameter were considered germinated.

### 2.3. Climate Conditions at the Main Cities of Soybean Production

The records of temperature (°C), relative humidity (%) (maximum and minimum), and rainfall (mm) for the largest soybean-producing municipalities in each region of Brazil were obtained from the website of the National Meteorological Institute in 2017 [39]. The cities were selected according to the Systematic Survey of Agricultural Production conducted by the Brazilian Institute of Geography and Statistics for soybean production [40]. The meteorological data were used to build graphics with the number of hours per day that the temperatures were in the ranges of <15 °C, 16–20 °C, 21–25 °C, 26–30 °C, 31–35 °C, and >35 °C for all months of 2017. The average daily relative humidity (%) for each month was added to the graph (Figure S1A–L). The number of hours per day that the temperatures were in the ranges described above never exceeded a 6 h period for the main municipal production regions.

### 2.4. Experimental Procedure at Different Temperature Ranges

Based on the graphics of the temperatures (Figure S1A–L) observed in the different regions of Brazil, temperatures ranging from 10 to 35 °C were chosen to conduct the experiments on fungal mycelial growth, conidial production, and virulence to nymphs. All experiments were carried out in incubators. Temperature and relative humidity were monitored at 1 h intervals by two dataloggers (Hobo^®^ U12-012, Onset Computer Corp. Ltd., Bourne, MA, USA) per incubator (Figures S2–S7). To maintain the temperature of each incubator with the lowest possible variation and ensure humidity between 60% and 70%, daily monitoring was conducted for five days prior to the experiments. To increase the humidity, when necessary, particularly at temperatures exceeding 30 °C, water containers were placed at various locations within the incubator until the desired standard was achieved.

### 2.5. Fungal Growth and Conidial Production at Different Temperatures and Hourly Intervals

The methodological information for the experiments on mycelial growth and conidial production of *C. javanica* on PDA is provided in Table 1.

For the experiments listed in Table 1, two microliters of a suspension at 1 × 10^7^ conidia mL^−1^ were placed in the center of Petri dishes (60 × 15 mm) containing 5 mL of PDA medium. Inoculated plates were incubated at temperatures described in Table 1. For all experiments, each treatment was replicated 10 times (1 Petri dish per treatment).

Fungal growth colonies (length and width of colonies in mm) were determined at days described in Table 1 using a digital caliper (150 mm/6” Digimess-100.174BL). Conidial production was determined after 10 days by cutting the fungal colony from the culture plate with a sterile scalpel. The colony disk was added in 30 mL of 0.01% Tween 80 and vortexed for five min at 700 rpm to dislodge conidia from the PDA. Successive dilutions were carried out until a desirable suspension was obtained for conidia counting using a Neubauer chamber (400× magnification). Conidial production was expressed as the number of conidia per colony.

### 2.6. Virulence of C. javanica to 2nd Instar Nymphs at Different Temperatures and Hourly Intervals 

Three experiments were conducted to determine the effect of different temperatures on the virulence of *C. javanica* to *B. tabaci* nymphs according to the methods described by Boaventura et al. [6] (Table 2).

Experimental units contained two 10-day-old common bean plants (*Phaseolus vulgaris*, cv. Pérola) grown in plastic pots filled with oxisol soil (2 L) and kept in a screenhouse (9 × 8 m) covered with a fine screen fabric (50 mesh). The experiments were carried out on common bean plants since soybean plants were highly sensitive to the elevated temperatures in the incubator. Adult-infested plants were shaken and placed near pest-free bean plants (10 days old with two primary leaves) for 6 to 8 h to allow oviposition. This procedure provided more than 100 eggs per leaf. The adult whiteflies were removed, and newly infested plants moved to another screenhouse until nymphs reached the 2nd instar (0.30– 0.44 mm in length and 0.18–0.36 mm in width) [41].

For all experiments, treatments were applied to the abaxial side of primary leaves containing 2nd instar nymphs with a microsprayer (0.3 mm needle, Paasche^®^ airbrush type H-set) connected to a vacuum pump and calibrated to 500 μL per leaf in an even coverage. Fungal treatments were sprayed with 2 × 10^7^ conidia mL^−1^. Controls consisted of nymphs sprayed with distilled water.

All experiments were conducted in a completely randomized design with four replicates, each consisting of three seedlings per pot (six primary leaves). One leaf from each pot was collected for nymphal mortality assessment at 3, 4, 5, 6, and 7 days after spraying and evaluated under a dissecting stereomicroscope at 40× magnification. A small red dot was marked with a permanent marker pen on the leaf next to the dead nymphs to confirm fungal infection. Nymphs that became desiccated or developed yellowish signs with mycelial or conidial growth on the insect cadaver were considered dead by *C. javanica*. The leaves were incubated inside gerbox-type boxes (11 × 11 × 3.5 cm) with wet cotton added to the leaf petiole for 4 days at room temperature (mean 26.1–30.2 °C, 53.0–73.7% RH). The dead marked nymphs presenting conidiogenesis (i.e., mycosed insects) were also considered infected by the fungus.

### 2.7. Modeling the Performance of C. javanica for Whitefly Control in Different Regions of Brazil

Temperature is considered the main climatic factor in the context of this work. To determine the performance of *C. javanica* at different temperature environments, several parameters were analyzed, including mycelial growth, conidial production, speed of insect mortality (LT_50_ value), and virulence of *C. javanica* to *B. tabaci* nymphs under temperatures ranging from 10 to 35 °C. Temperature data were obtained from the WorldClim database (http://www.worldclim.org/, accessed on 14 March 2023). These datasets are made of layers containing monthly mean, minimum, and maximum records in raster files in “Flat” format (.flt and.hdr file), with a spatial resolution varying from 10 arc-minutes (~20 km) to 30 arc-seconds (~1 km). The 30 arc-seconds (~1 km) were used in the context of this study.

An open-source computer-aided tool built on R codes and Java interface, the entomopathogenic fungi application (EPFA) software version 1.045, was used for modeling the virulence of *C. javanica* against *B. tabaci*. The recorded mortality was plotted against the corresponding temperature values from which nonlinear models were fitted to the observed data [42]. The model parameters were estimated by fitting equations to the recorded mortality and the corresponding temperature values. First, 82 models were fitted to the data; then, the best-fitted models were selected based on their coefficient of determination (R^2^), adjusted R^2^, Akaike’s information criteria, the root mean square error, and residual sum of squares (RSS) [42].

The temperature-dependent mathematical expression obtained during the modeling step was run at each grid of the raster files of Brazil using the monthly minimum and maximum temperature datasets obtained from WorldClim. The gridded temperature datasets were loaded into EPFA software, simultaneously extracted from the database, and then organized in matrix format using longitude as the columns and latitude as the rows [42]. The monthly temperature data from Brazil, in raster format as required by the EPFA software, are from 2018, which were the most recent data available. A point object picks the temperature-dependent mathematical expression of the fungal virulence, which was consecutively applied in each geographical coordinate of the grid. The results were converted into ASCII file format (.asc) and transferred into an open-source software Q-GIS60 for visualization [42]. The virulence map was produced for Brazil after completing the fitting process. Details of procedures and steps to estimate the best model parameters as per the defined input data can be found in Guimapi et al. [42].

### 2.8. Data Analysis

For mycelial growth and virulence experiments, curves were adjusted according to nonlinear models (Logistic, Log Logistic, Log-normal, Weibull, and Gompertz) and compared using the Chi-square test (*p* < 0.05). This nonparametric statistical method was used for the comparison of two unpaired groups to verify whether or not they belong to the same population and when the requirements for the application of the Student’s *t*-test were not met.

To estimate the median lethal time (LT_50_), nonlinear models were fitted, and values were compared by the overlap of their 95% confidence intervals (95% CI) using the package “drc 3.0-1” [43]. The LT_50_ was not estimated for treatments where mortality did not reach 50%. For conidial production, data were submitted to analysis of variance, and a multiple means comparison was made using Tukey’s HSD test (*p* < 0.05).

Nonlinear models were used to determine the optimal temperature for mycelial growth and virulence of *C. javanica* to *B. tabaci*. The best-fitted model was selected based on the goodness-of-fit of the model using the coefficient for nonlinear regression (R^2^) and the RSS. Higher R^2^ values and lower RSS values suggest a better fit [44]. The models and equations used to describe optimal temperatures are shown in Table 3.

In all the model equations described in Table 3, $r_{T}$ represents the fungal development rate and *T* (°C) is the observed temperature. $T_{1}$ and $T_{2}$ are the minimum and maximum temperatures, respectively, at which the growth rate is zero. The $\sqrt{c_{c}}\;k_{1}$ and $c_{c}\;$ correspond to the slope of the regression as described in the rootsq_82 equation, and k and $k_{2}$ are constants [45,46,47,48]. These expressions are included in the “devRate” package [available at https://cran.r-project.org/web/packages/devRate/devRate.pdf, accessed on 27 February 2023].

## 3. Results

### 3.1. Fungal Mycelial Growth

**Figure 1:**
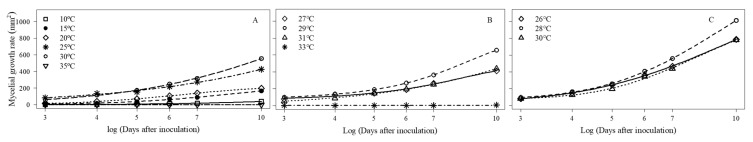
The mean diameter of *Cordyceps javanica* colonies at different constant temperatures for 10 days. Curves were adjusted according to nonlinear models: Log logistic (**A**), Logistic (**B**), and Log-normal (**C**).

Mycelial growth of *C. javanica* occurred at all temperatures except 35 °C (Experiment 1) (Figure 1A). However, the mean diameter of colonies was significantly slower and reduced at 10, 15, and 20 °C (38.9 to 196.4 mm^2^) compared to 25 and 30 °C (425.7 and 554.4 mm^2^, respectively) (Figure 1A, Table 4).

In the second experiment, *C. javanica* colony growth was significantly higher at 29 °C (655.6 mm^2^) than at 27 °C (411.8 mm^2^) and 31 °C (439.1 mm^2^) for 10 days (Figure 1B, Table 4). No fungal growth was observed at 33 °C (Figure 1B). In Experiment 3, the mean diameter of colonies was similar at 26, 28, and 30 °C (779.2, 1013.4, and 787.3 mm^2^, respectively) for 10 days (Figure 1C, Table 4).

**Figure 2:**
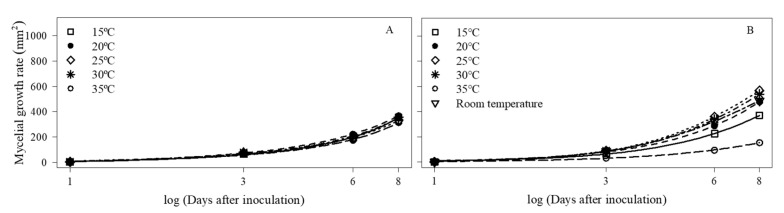
The mean diameter of *Cordyceps javanica* colonies after exposure to different temperatures, followed by mean temperatures of 27.4 and 28.5 °C for 10 days. (**A**) = 6 h at each temperature before being transferred to the mean temperature of 27.4 °C (Experiment 4). (**B**) = 6 h alternating with 18 h at fluctuating temperatures (mean of 28.5 °C) (Experiment 5). Curves were adjusted according to nonlinear models: Logistic (**A**) and Log Logistic (**B**).

No difference in colony growth was observed when the fungus was held for 6 h at 15, 20, 25, 30, or 35 °C and then transferred to 27.4 °C for 8 days (Figure 2A, Table 5). When the fungus was held for 6 h at 15, 20, 25, 30, or 35 °C, alternating with 18 h at 28.5 °C for 8 days, mycelial growth occurred at all temperatures. However, the growth of colonies was slower and reduced at 35 °C (152.9 mm^2^) compared to 20, 25, and 30 °C and 28.5 °C (476.6 to 565.9 mm^2^) for 8 days (Figure 2B, Table 5).

**Figure 3:**
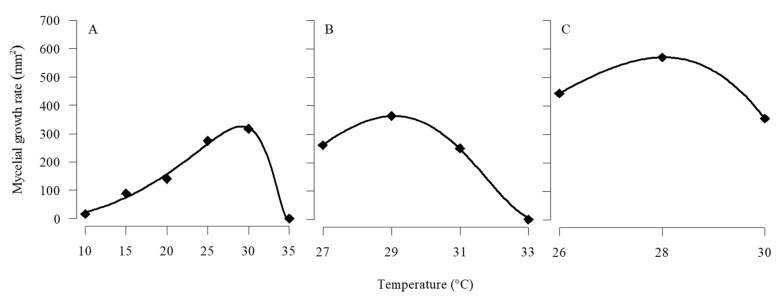
Relationship between temperature and mycelial growth rate of *C. javanica*. Nonlinear models fitted to observed values of the mycelial growth rate of *C. javanica* at constant temperatures of (**A**) 10, 15, 20, 25, 30, and 35 °C (Experiment 1); (**B**) 27, 29, 31, and 33 °C (Experiment 2); and (**C**) 26, 28, and 30 °C (Experiment 3).

The optimal temperature for *C. javanica* mycelial growth was estimated at 28.0 to 29.1 °C based on parameters from the nonlinear Ratkowsky 1 and Ratkowsky 2 models fitted to the mean diameter of colonies versus temperature (Figure 3). Statistics of the goodness-of-fit of models describing the relationship of temperature with the mean diameter of *C. javanica* colonies are presented in Table 6.

### 3.2. Conidial Production on PDA Medium

At constant temperatures, fungal conidial production at 15 and 20 °C was 0.8 and 2.7 × 10^6^ conidia colony^−1^, respectively, and significantly lower than 25 °C (100 × 10^6^ conidia colony^−1^) and 30 °C (220 × 10^6^ conidia colony^−1^) after 10 days (Table 7). There was no conidial production at 10 and 35 °C because there was no mycelial growth (Table 7).

When the fungus was held for 6 h at 15, 20, 25, 30, or 35 °C before being transferred to 27.4 °C for 10 days, conidial production at 35 °C (193.9 × 10^6^ conidia colony^−1^) was significantly higher than at 20 °C (78.2 × 10^6^ conidia colony^−1^). However, no differences were observed for 15, 25, and 30 °C compared to 20 and 35 °C (Table 7).

On the other hand, when *C. javanica* was incubated for 6 h at 15, 20, 25, 30, or 35 °C, alternating with 18 h at 28.5 °C for 10 days, conidial production at 25 and 30 °C (146.2 and 160.8 × 10^6^ conidia colony^−1^, respectively) were significantly higher than at 15 °C (32.5 × 10^6^ conidia colony^−1^) and 35 °C (48.7 × 10^6^ conidia colony^−1^). No differences in sporulation were observed at 20 °C compared to other temperatures tested, except at 15 °C (Table 7).

### 3.3. Virulence of C. javanica to 2nd Instar Nymphs at Different Temperatures and Hourly Intervals

**Figure 4:**
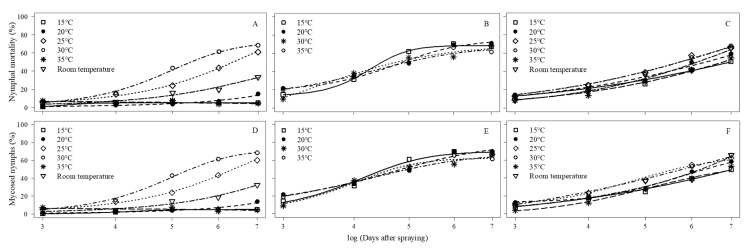
Nymphal mortality (**A**–**C**) and mycosed nymphs (**D**–**F**) on different days post-inoculation of 2nd instar *B. tabaci* with *C. javanica* after exposure to different temperatures for 7 days. (**A**,**D**) = Constant temperatures for 7 days (Experiment 1). (**B**,**E**) = 6 h at each temperature before being transferred to fluctuating temperatures (mean of 28.4 °C) for 7 days (Experiment 2). (**C**,**F**) = 6 h at 15, 20, 25, 30, or 35 °C, alternating with 18 h at fluctuating temperatures (mean of 30.2 °C) for 7 days (Experiment 3). Curves were adjusted according to nonlinear Logistic (**A**,**C**,**F**), Weibull (**B**,**D**), and Gompertz (**E**) models.

At constant temperatures for 7 days, nymphal mortality at 15, 20, and 25 °C was very low (5.3–14.9%) and significantly slower than for 25 and 30 °C (60.9% and 68.5%, respectively) (Figure 4A, Table 8). At 25 and 30 °C, mortality was significantly higher than the untreated control (*p* < 0.001). The fungus killed faster at 30 °C (LT_50_ 5.4 days) compared to 25 °C (6.3 days) (Table 9).

No difference in nymphal mortality was observed when the fungus was held for 6 h at 15, 20, 30, and 35 °C before being transferred to 26.1 °C (min. 24.3 and max. 28.9 °C) for 7 days (Figure 4B, Table 8). Fungal treatments killed significantly more nymphs than the untreated control at all temperatures (*p* < 0.001). The LT_50_ values at 15 °C (4.5 days) were significantly different from those at 20 °C and 35 °C (4.9 days for both), but the mortality at all temperatures was similar to 30 °C (4.7 days) (Table 9).

There was no difference in nymphal mortality among the fungus treatments (Figure 4C, Table 8) when the treated nymphs were held for 6 h at temperatures of 15, 20, 25, 30, or 35 °C, alternating with 18 h at 30.2 °C (min. 26.5 and max. 34.4 °C) for 7 days. The nymphal mortality was significantly higher when compared to the untreated control (*p* < 0.001). The speed of infection of *C. javanica* was faster at 25 and 30 °C than at 15, 20, 35, and 28.4 °C. The LT_50_ was 5.7 days at both 25 and 30 °C, while it ranged from 6.11 to 6.94 days under the other temperature conditions (Table 9).

For all three experiments, nymphal mortalities were very similar to the percentage of cadavers with fungal sporulation (i.e., mycosed nymphs) at all temperatures tested (Figure 4D–F). The *p*-values of the comparisons of confirmed mortality curves for *B. tabaci* nymphs are also reported as a Supplementary File (Table S1).

**Figure 5:**
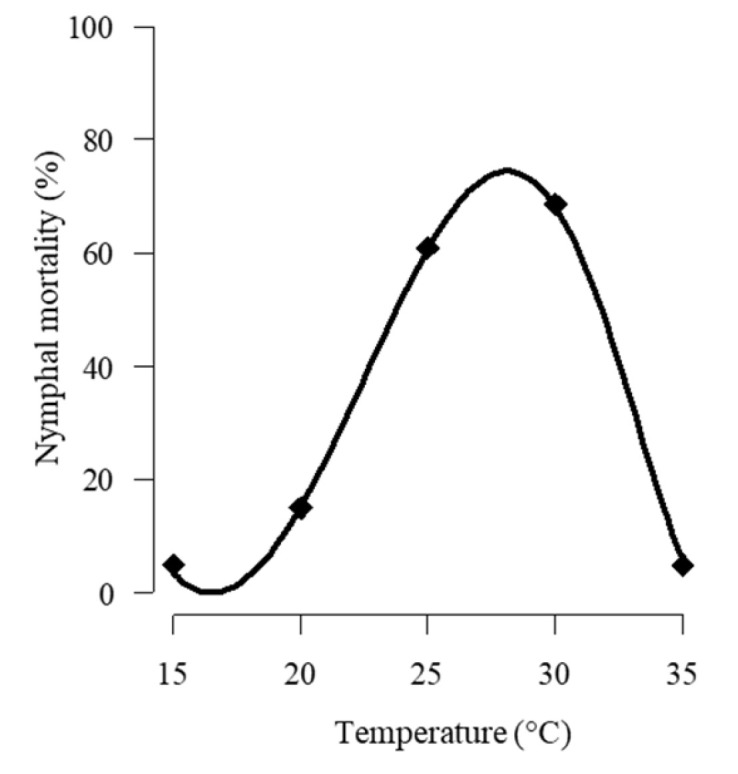
Graphical comparison of a temperature-dependent model describing nymphal mortality of *C. javanica* after constant temperature. The curve represents the Ratkowsky 1 nonlinear model that best fitted the experimental data points and was used to predict the virulence of the fungus against the whitefly. (Parameters: $c_{c}\;$ = 2.999, $k_{1}\;$= 0.084, $k_{2}\;$= 0.090,$\;T_{1}\;$= 16.502, $T_{2}\;$= 36.051; R^2^: 0.959, RSS: 0.067).

The Ratkowsky 1 model had a good fit to the mortality data, and the model adjusted for the mortality versus temperature is shown in Figure 5. The nonlinear model predicted an optimal temperature for higher mortality of *B. tabaci* nymphs by *C. javanica* at 28.1 °C.

### 3.4. Spatial Prediction of the Performance of C. javanica Against B. tabaci at Different Regions of Brazil 

**Figure 6:**
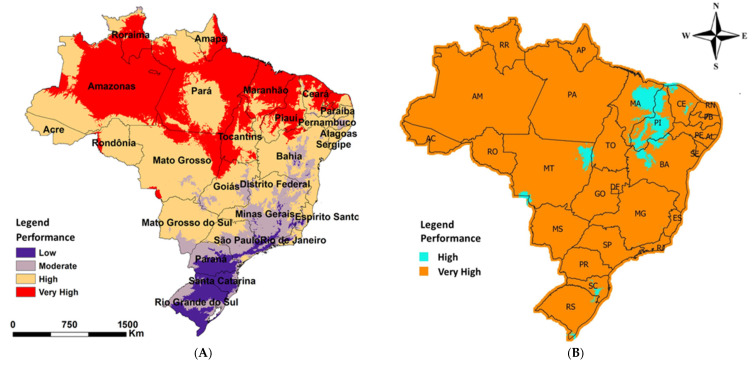
A spatial model of the predicted performance of Lalguard C99 for the control of *B. tabaci* in Brazil according to the virulence experiments at constant temperature for 7 days (**A**), 6 h only, or for 6 h daily at 15, 20, 25, 30, and 35 °C followed by 7 days of fluctuating temperatures (mean of 28.4–30.2 °C) for 7 days (**B**). The figures were generated using QGIS 3.16 software. The names in the figure are the Brazilian states.

The predicted performance of the bioinsecticide Lalguard C99 against *B. tabaci* biotype B in Brazil is shown in Figure 6. The model based on constant temperatures predicted that the application of *C. javanica* in Brazil would result in high performance (more than 60% mortality of *B. tabaci*) in the country’s northern, northeastern, and central–western regions and low to moderate performance (10–25% mortality) for southern and southeastern regions (Figure 6A). However, when *C. javanica* was exposed to different temperatures for 6 h only or for 6 h daily followed by 7 days of fluctuating temperatures (mean of 28.4–30.2 °C), the model predicted a high performance of Lalguard C99 (over 60% mortality in *B. tabaci*) across all regions of Brazil (Figure 6B).

## 4. Discussion

The majority of bioassays designed to assess the development and virulence of entomopathogenic fungi (EPF), including *Cordyceps* sp., have been conducted in vitro at constant temperature regimes [33,36,49,50,51,52,53]. These studies have indicated that the optimum temperature for vegetative growth of *Cordyceps* sp. ranged from 25 to 30 °C, with growth being delayed at 15 °C and completely inhibited at 35 °C. These findings are consistent with our study under constant temperatures, where we observed that the ideal temperature range for the growth of *C. javanica* was between 25 and 30 °C, with optimal growth occurring between 28 and 29.1 °C. In addition, at constant temperatures, conidial production was higher at 25 and 30 °C and reduced at 15 and 20, and no production occurred at 35 °C. Xie et al. [54] reported that conidial production of *C. javanica* Pf04 on rice was higher at 25 °C than at 30 °C, with reduced production at 20 °C. However, in previous studies on rice, *C. javanica* BRM27666 also showed higher conidial production at 30 °C after 10 days [Quintela et al., unpublished data], suggesting that this strain has a higher optimal sporulation temperature. This highlights significant variation in temperature tolerance among strains [36].

Temperatures are not constant under field conditions. After field applications, conidia may be exposed to fluctuating temperatures, which could negatively impact their viability and virulence. In the field, temperatures fluctuate both throughout the year and between day and night. In this study, we created graphs that illustrate the number of hours that temperatures remain within specific ranges over the course of the year. For example, during the summer season, temperatures fluctuate significantly and can remain above 35 °C or below 15 °C for about 6 h, depending on the region. To simulate this effect, we first exposed the fungus in vitro to temperatures of 15, 20, 25, 30, and 35 °C for 6 h only, followed by 27.4 °C over the course of 10 days. Our study revealed that during the initial stages of germination, these temperatures did not affect mycelial growth on PDA. In addition, daily exposure to constant temperatures of 15, 20, 25, 30, and 35 °C for 6 h on a PDA medium, alternating with 18 h of fluctuating temperatures (mean of 28.5 °C) over a period of 8 days, reduced mycelial growth at the upper-temperature limit of 35 °C. This likely occurred because hyphae are less tolerant to high temperatures than conidia, which are naturally more heat-resistant structures [55,56,57,58]. Hyphal growth takes place inside the host, where the hyphae benefit from a degree of protection against hostile environmental conditions.

The infection process by EPF initiates with conidia germination and penetration in the insect cuticle and consequently develops into hyphae and mycelia in the hemocoel, effectively colonizing the insect’s body [59,60,61]. After the fungus invades and kills the host, it produces new conidia under favorable temperature and high humidity conditions, which are essential for disease transmission to other hosts and maintaining its inoculum in the environment [62,63,64]. When our fungus was exposed to 15, 20, 25, 30, or 35 °C for 6 h only, followed by 10 days at 27.4 °C, conidial production was not affected. However, after exposure for 6 h daily at these temperatures followed by 10 days at room fluctuating temperatures (mean of 28.5 °C), the highest conidial production (≈10^8^ conidia/colony) occurred at 20, 25, and 30 °C, while production at 15 and 35 °C was slightly lower (≈10^7^ conidia/colony). These results show that probably six hours daily in regions with high-temperature regimes in Central, Northern, and Northeastern Brazil will reduce conidial production on the cadavers. In Southern and Southeastern Brazil, conidia production will be reduced only at temperatures below 15 °C.

In addition to our in vitro studies using artificial culture media, we also conducted in vivo studies with second instar whitefly nymphs, *B. tabaci*. At constant temperatures, the mortality rates of whitefly nymphs by *C. javanica* were low at 15, 20, and 35 °C (range of 5.3 to 14.9%). The highest level of nymphal mortality was observed at 25 and 30 °C (range of 60.9 to 68.5%), exhibiting a lower LT_50_ at 30 (5.4 days) compared to 25 °C (6.3 days). When second instar nymphs were exposed to the fungus for 6 h only or 6 h daily at different temperatures followed by seven days at fluctuating temperatures (mean of 28.4–30.2 °C), nymphal mortality was similar for all temperatures, and small differences were observed for LT_50_ values. This indicates that, after penetration, the development of the fungus within the insect is not influenced by temperature.

Cabanillas et al. [33] observed that the mycelial growth of *Cordyceps* sp. on PDA was higher at 30 °C than at 25 °C, indicating that this fungus has a higher optimal growth temperature than many other EPF. Fargues et al. [34] reported that the optimal temperature for growth is not necessarily the same as that for fungal infection of insects. On the other hand, Moorehouse et al. [65] showed that EPF tends to kill the host faster at temperatures that are optimum for fungal mycelial growth. In our study, fungal growth and infection of *B. tabaci* nymphs by *C. javanica* were similar at a temperature range of 25 to 30 °C, and the optimum temperature was between 28 and 29.1 °C. Furthermore, constant temperatures outside this range affected the development of the fungus both in the PDA medium and in the insect host. For this reason, we also tested the virulence of *C. javanica* to whitefly nymphs under fluctuating temperature conditions.

In the summer season, field temperatures often exceed 35 °C. However, these high temperatures for relatively short periods did not affect its virulence against whitefly nymphs, as observed in this study. On the insect’s cuticle, fungal conidia typically invade the bodies of their hosts within 24 h [20]. After this period, most of the conidia in the field are lost, mainly due to exposure to solar radiation, high temperatures, and precipitation. Studies have shown that most *C. javanica* conidia lose viability in the field within 24 h after application due to the incidence of solar radiation [52] [Quintela et al., unpublished data]. Thus, the initial 24 h after the conidium contacts the insect’s cuticle is the most critical period for the fungus to infect the host. Therefore, we strongly recommend applying *C. javanica* in the late afternoon in the field because there is an interaction between solar radiation and temperature in the persistence of conidia, where higher temperature aggravates the detrimental effect of solar radiation on conidial viability [66]. This approach is particularly important when managing mobile insects, such as whitefly adults or corn leafhoppers, which may come into contact with the conidia on the leaf after application.

Whitefly outbreaks typically occur at high temperatures during the summer crop season, demonstrating that the fungus can be used to control this pest when temperatures > 35 °C do not exceed 6 h per day. Under extreme temperatures during the growing season, the fungus, insects, and plants may be adversely affected [67]. Temperature has profound effects on the physiology and development of both the insect host and fungal pathogen (which simultaneously affect host susceptibility and pathogen virulence), and these effects are influenced, in turn, by factors related to the host plant. Evapotranspiration, for example, can lower leaf surface temperatures to levels substantially below ambient [68,69]. In the same way that high temperatures (35 °C) for prolonged periods affect the growth of the fungus, these conditions can influence the survival of *B. tabaci* and the development of soybean plants. Low temperatures also limit the growth and development of both whitefly and soybeans, similar to the fungus [70,71,72].

Mathematical models have become an important tool for understanding and predicting the virulence and suitable application areas for entomopathogenic fungi against insect pests often occurring in different environmental conditions [42,73]. Considering the virulence data under constant temperature, spatial prediction revealed that in some regions of Brazil, the fungus may effectively control whiteflies, while in other areas, the performance may be very low. For example, in the northern, northeastern, and central–western regions, the models predicted a higher virulence of *C. javanica*, while low to moderate virulence was predicted in the southern and southeastern regions of the country. However, the lowest temperatures observed in these regions of the country generally occur between May and July when whitefly populations are smaller [10]. Furthermore, soybeans are not usually cultivated during this period [74]. The temperature-dependent modeling was carried out considering the soybean growing season in Brazil. Based on these conditions, the spatial prediction revealed that *C. javanica* could be highly virulent to *B. tabaci* in all regions of Brazil. Thus, even in tropical soybean-producing regions, where higher average temperatures are recorded (>35 °C), such as a large part of MATOPIBA (states of Maranhão, Tocantins, Piauí, and Bahia) and the central–west region (states of Mato Grosso, Mato Grosso do Sul, and Goiás), the Lalguard C99 mycoinsecticide could be an excellent tool for managing *B. tabaci* populations. In the southern and southeastern regions, average temperatures remain close to the optimal temperature range (25 to 30 °C) for *C. javanica*, which suggests the product’s high effectiveness.

Agbessenou et al. [75] used constant temperatures to establish temperature-dependent models to identify geographically suitable areas for deploying *M. anisopliae* for *Tuta absoluta* (Meyrick, 1917) control in East Africa. Their study revealed that the fungal performance was location-specific. Similarly, when we modeled our data to develop spatial predictions of potential areas for *C. javanica* using constant temperatures, Brazil was divided into regions with low, moderate, high, and very high performance. However, when we used fluctuating temperatures (more representative of field conditions), our results showed that the fungus can be used across all regions of Brazil with high to very high performance. Our spatial prediction results indicate that Lalguard C99 could become a key tool for reducing *B. tabaci* biotype B populations in the field, both at low densities (during the initial whitefly infestations from November to December) and at high densities with overlapping generations (from January to March) under field conditions. The environmental conditions, including temperature and high humidity due to the rainy season, support the efficacy of Lalguard C99 for whitefly control across Brazil.

Other factors can also be considered that will guarantee the effectiveness of Lalguard C99 in controlling whiteflies in soybeans. In previous studies, Boaventura et al. [6] observed that temperature was more detrimental to *C. javanica* virulence against *Bemisia* nymphs than relative humidity. This is probably because many pathogens find sufficient moisture for germination and host penetration within the leaf or insect microclimate boundary layer [29,30]. However, to promote horizontal transmission in whitefly populations that lead to epizootics, widespread infection of *C. javanica* must occur under high humidity conditions, ideal temperatures (25–30 °C), frequent rainfall, and high host densities [76]. *C. javanica* BRM27666 was isolated from a whitefly adult from epizootic on soybeans in Central–Western Brazil. Therefore, in addition to its greater tolerance to high temperatures, probably linked to its geographic origin [36], this strain is also highly adapted to soybean cultivation conditions. Another factor that may benefit the development of epizootics caused by *C. javanica* in whitefly populations in soybean is the microclimate conditions achieved by dew formation, creating a humid environment near the leaf surface. When plants are sown with 45 cm between rows, dew formation reaches between 1.000 and 1.500 cm^2^ of leaf area [31], and this microclimate is excellent for promoting fungal development.

Soybean is the main crop that hosts whiteflies as well as one of the main commodities grown in Brazil [11,77]. Therefore, the spatial prediction of suitable areas for the use of Lalguard C99 in this study was based on climatic conditions, mainly temperature, observed during the soybean growing season in the main producing regions of the country. For other economically important crops that are also highly affected by *B. tabaci*, the climatic conditions during each growing season must be considered. For example, *B. tabaci* is one of the main pests affecting melon crops, particularly concentrated in the northeastern region in the states of Ceará and Rio Grande do Norte. In these regions, melon is cultivated throughout the year due to favorable climatic conditions, including high temperatures (ranging from 25 to 35 °C), low relative air humidity, and long periods of exposure to the sun, in addition to minimal rainfall, which is less of a limiting factor in production [78,79,80].

## 5. Conclusions

Our study provides a comprehensive understanding of the effects of both constant and fluctuating temperatures on the growth, conidial production, and virulence of *C. javanica* against *B. tabaci*. While constant temperatures clearly delineate the optimal range for fungal growth and infection (25–30 °C), fluctuating temperatures that replicate field conditions do not compromise fungal virulence, even when exposed to extreme temperatures (15 °C and 35 °C) for 6 h daily. However, it is important to recognize that the success of fungal infection is influenced by other factors, including solar radiation, humidity, rainfall, the insect’s immune response, and its associated microbiota. Considering the soybean growing season in Brazil, temperature-dependent models predicted that the bioinsecticide Lalguard C99 BRM27666 will be effective against *B. tabaci* in all Brazilian regions based on its efficacy across a wide range of temperature regimes (for growth and conidiogenesis), speed of kill (LT_50_), and virulence against this pest. The results of this study replicate field conditions, as Lalguard C99 has demonstrated high efficacy in controlling whiteflies in commercial areas in all Brazilian regions. To further support the findings of our research, monthly experiments are being conducted under varying solar radiation, temperature, and moisture conditions.

## Figures and Tables

**Table 1:** Temperatures and assessment days for mycelial growth and conidial production of *C. javanica* on PDA medium. A 6 h period was chosen since the temperature ranges of <15 °C, 16–20 °C, 21–25 °C, 26–30 °C, 31–35 °C, and >35 °C never exceeded 6 h daily during the soybean cultivation season (October through March) [39].

Experiment	Temperature (°C)	Exposure Time	Assessment Days	
			Radial Growth	Conidial Production
1	10, 15, 20, 25, 30, 35	24 h for 10 days	3, 4, 5, 6, 7, 10	10
2	25, 27, 29, 31, 33	24 h for 10 days	3, 4, 5, 6, 7, 10	Not determined
3	26, 28, 30	24 h for 17 days	3, 4, 5, 6, 7, 10, 11, 12, 13, 14, 17	Not determined
4	15, 20, 25, 30, 35	6 h at each temperature and then 27.4 °C for 10 days ^1^	1, 3, 6, 8	10
5	15, 20, 25, 30, 35, 28.5 °C	6 h alternating with 18 h at 28.5 °C for 10 days ^2^	1, 3, 6, 8	10

^1^ Mean: 27.4 °C; min: 27.0 °C; max: 29.9 °C; RH: between 60 to 70%. ^2^ Room temperature: mean 28.5 °C; min: 26.4 °C; max: 31.6 °C; RH: between 60 to 70%.

**Table 2:** Temperatures and assessment days for virulence of *C. javanica* against 2nd instar nymphs of *B. tabaci*.

Experiment	Temperature (°C)	Exposure Time	Mortality (Assessment Days)
1	15, 20, 25, 30, 35, 28.4 °C ^1^	24 h	3, 4, 5, 6, and 7
2	15, 20, 30, 35	6 h at each temperature and then 28.4 °C	
3	15, 20, 25, 30, 35, 30.2 °C ^2^	6 h alternating with 18 h at 30.2 °C	

^1^ Room temperature: mean: 28.4 °C; min: 25.2 °C; max: 32.4 °C; RH: between 60 to 70%. ^2^ Room temperature: mean: 30.2 °C; min: 26.5 °C; max: 34.4 °C; RH: between 60 to 70%.

**Table 3:** Mathematical equations describing the relationship between temperature and colony growth (Experiments 1, 2, and 3) and virulence (Experiment 1) of *C. javanica* conducted at constant temperatures. For models, $r_{T}$ represents the fungal development rate, T is the observed temperature (°C), $T_{1}$ and $T_{2}$ are the minimum and maximum temperatures, $\sqrt{c_{c}}k_{1}\;and\;c_{c}$ correspond to the regression slope, and k and $k_{2}$ are constants.

Experiments	Model	Mathematical Expressions	Model Parameters
Colony Growth	Ratkowsky 1 ^1^	$r_{T}=\sqrt{c_{c}}k_{1}\left(T-T_{1}\right)(1-\exp\left[k_{2}\left(T-T_{2}\right)\right]){)}^{2}$	$\sqrt{c_{c}}k_{1},\;k_{2},\;T_{1},\;T_{2}$
	Ratkowsky 2 ^2,3^	$r_{T}=(c_{c}(T-T_{1})(1-\exp\left[k\left(T-T_{2}\right)\right]){)}^{2}$	$c_{c},\;k,\;T_{1},\;T_{2}$
Virulence	Ratkowsky 1 ^4^	$r_{T}=\sqrt{c_{c}}k_{1}\left(T-T_{1}\right)(1-\exp\left[k_{2}\left(T-T_{2}\right)\right]){)}^{2}$	$\sqrt{c_{c}}k_{1},k_{2},\;T_{1},\;T_{2}$

^1^ Experiment 1: 10, 15, 20, 25, 30, and 35 °C; ^2^ Experiment 2: 27, 29, 30, and 31 °C; ^3^ Experiment 3: 26, 28, and 30 °C). ^4^ Experiment 1: 15, 20, 25, 30, and 35 °C.

**Table 4:** *p*-values (*p* ≤ value) of the comparisons of mycelial growth curves of *C. javanica* at different temperatures. Curves were considered significantly different at *p* ≤ 0.05. Experiment 1, 2, and 3 = constant temperatures for 10 days.

Mycelial Growth					
Experiment 1					
Temperature (°C)	10	15	20	25	30
15	0.220	-	-	-	-
20	0.068	0.527	-	-	-
25	<0.001	0.004	0.022	-	-
30	<0.001	<0.001	0.001	0.281	-
35	0.735	0.118	0.031	<0.001	<0.001
Experiment 2					
Temperature (°C)	27		29	31	
29	0.034		-	-	
31	0.845		0.049	-	
33	<0.001		<0.001	<0.001	
Experiment 3					
Temperature (°C)	26			28	
28	0.338			-	
30	0.836			0.298	

**Table 5:** *p*-values (*p* ≤ value) of the comparisons of mycelial growth curves of *C. javanica* at different temperatures. Experiment 4 = 6 h at each temperature before being transferred to an incubator (mean of 27.4 °C). Experiment 5 = 6 h alternating with 18 h at fluctuating temperatures (mean of 28.5 °C). Curves were considered significantly different at *p* ≤ 0.05.

Mycelial Growth					
Experiment 4					
Temperature (°C)	15	20	25		30
20	0.852	-	-		-
25	0.941	0.832	-		-
30	0.942	0.901	0.924		-
35	0.868	0.739	0.902		0.829
Experiment 5					
Temperature (°C)	15	20	25	30	35
20	0.378	-	-	-	-
25	0.077	0.374	-	-	-
30	0.134	0.536	0.786	-	-
35	0.072	0.007	<0.001	<0.001	-
28.5	0.227	0.726	0.566	0.755	0.003

**Table 6:** Estimated parameters for nonlinear Ratkowsky models 1 and 2 describing the relationship between temperature and mean diameter of *C. javanica* colonies for Experiments 1 to 3 conducted at constant temperatures. R^2^ and RSS were used to select the best-fitted model.

Model	Parameters	Experiments ^1^		
		1	2	3
Ratkowsky 1	$c_{c}$	0.089	-	-
	$k_{1}$	2.620	-	-
	$k_{2}$	0.435	-	-
	$T_{1}$	3.926	-	-
	$T_{2}$	34.845	-	-
Ratkowsky 2	$c_{c}$	-	5.919	3.670
	k	-	0.177	0.410
	$T_{1}$	-	22.907	19.634
	$T_{2}$	-	33.250	31.671
R^2^		0.972	0.911	0.873
RSS		2473	7009	1424

^1^ Experiment 1: 10, 15, 20, 25, 30, and 35 °C; Experiment 2: 27, 29, 30, and 31 °C; Experiment 3: 26, 28, and 30 °C.

**Table 7:** Conidial production of *C. javanica* after exposure for 10 days to constant temperatures (Experiment 1), 6 h at 15, 20, 25, 30, or 35 °C and then at the incubator (mean of 27.4 °C) for 8 days (Experiment 4), and 6 h at 15, 20, 25, 30, or 35 °C, alternating with 18 h at fluctuating temperatures (mean of 28.5 °C) for 8 days (Experiment 5).

Number of Conidia × 10^6^/Colony			
Temperature (°C)	Experiment 1	Experiment 4	Experiment 5
10	0.0 ± 0.0 c	- *	-
15	0.8 ± 0.7 b	118.9 ± 79.4 ab	32.5 ± 7.7 c
20	2.7 ± 2.0 b	78.2 ± 41.5 b	110.0 ± 41.4 ab
25	100.0 ± 180.0 a	162.8 ± 99.2 ab	146.2 ± 54.2 a
30	220.0 ± 90.2 a	120.8 ± 63.8 ab	160.8 ± 57.1 a
35	0.0 ± 0.0 c	193.9 ± 67.5 a	48.7 ± 57.4 bc

* This temperature was not tested. Each value is expressed as mean ± SD. Different lowercase letters show significant differences (Tukey’s HSD test) in sporulation among temperature regimes. Experiment 1 (F = 143.60; df = 4,45; *p* < 0.001); Experiment 4 (F = 2.67; df = 4,32; *p* = 0.049); Experiment 5 (F = 13.23; df = 4,41; *p* < 0.001).

**Table 8:** *p*-values (*p* ≤ value) of the comparisons of nymphal mortality curves for *B. tabaci* after treatment with *C. javanica* at different temperatures. Curves were considered significantly different at *p* ≤ 0.05. Experiment 1 = Constant temperatures for 7 days. Experiment 2 = 6 h at each temperature before being transferred to fluctuating temperatures (mean of 28.4 °C) for 7 days. Experiment 3 = 6 h at 15, 20, 25, 30, or 35 °C alternating with 18 h at fluctuating temperatures (mean of 30.2 °C) for 7 days. Curves were adjusted according to nonlinear Logistic (A, C) and Weibull models (B).

Nymphal Mortality					
Experiment 1					
Temperature (°C)	15	20	25	30	35
20	0.752	-	-	-	-
25	0.004	0.007	-	-	-
30	<0.001	<0.001	0.210	-	-
35	0.869	0.677	0.004	<0.001	-
28.4 *	0.181	0.255	0.119	0.006	0.172
Experiment 2					
Temperature (°C)	15	20		30	
20	0.552	-		-	
30	0.524	0.637		-	
35	0.414	0.628		0.781	
Experiment 3					
Temperature (°C)	15	20	25	30	35
20	0.685	-	-	-	-
25	0.307	0.535	-	-	-
30	0.318	0.550	0.980	-	-
35	0.770	0.588	0.253	0.262	-
30.2 *	0.541	0.728	0.500	0.510	0.568

* Average room temperature after seven days.

**Table 9:** LT_50_ values 7 days post-inoculation of whitefly nymphs treated with *C. javanica.* Experiment 1 = Constant temperatures for 7 days. Experiment 2 = 6 h at each temperature before being transferred to fluctuating temperatures (mean of 28.4 °C) for 7 days. Experiment 3 = 6 h at 15, 20, 25, 30, and 35 °C, alternating with 18 h at fluctuating temperatures (mean of 30.2 °C) for 7 days. Dash (-) means the LT_50_ value was not estimated for cumulative mortality less than 50% at 7 days post-treatment.

Temperature	LT_50_ (d) (CI95%)		
	Experiment 1	Experiment 2	Experiment 3
15	-	4.5 (4.4–4.5)	6.9 (6.8–7.0)
20	-	4.9 (4.7–5.0)	6.3 (6.3–6.4)
25	6.3 (6.1–6.6)	Not determined *	5.7 (5.6–5.7)
30	5.4 (5.3–5.4)	4.7 (3.8–5.7)	5.7 (5.6–5.7)
35	-	4.9 (4.6–5.3)	6.7 (6.7–6.8)
Room temperature	-	Not determined *	6.1 (6.1–6.2)

* Treatment not evaluated due to material loss.

## Data Availability

The original contributions presented in this study are included in the article/Supplementary Materials. Further inquiries can be directed to the corresponding authors.

## Supplementary Materials

The following supporting information can be downloaded at https://www.mdpi.com/article/10.3390/jof11020125/s1. Figure S1: Number of hours (represented by bars) that temperatures remain within the ranges of <15 °C, 16–20 °C, 21–25 °C, 26–30 °C, 31–35 °C, and >35 °C, and relative humidity (illustrated by a blue line), for the months of January through December 2017 in Brazil for the city and state of Vilhena, Rondônia (A); Rio Verde, Goiás (B); Sorriso, Mato Grosso (C); Formoso do Araguaia, Tocantins (D); Balsas, Maranhão (E); Bom Jesus, Piauí (F); Luis Eduardo Magalhães, Bahia (G); Castro, Paraná (H); Itapeva, São Paulo (I); Tupanciretã, Rio Grande do Sul (J); Unaí, Minas Gerais (K); and Brasília, Distrito Federal (L); Figure S2: Temperature (°C) and relative humidity (%) recorded at one-hour intervals during the mycelial growth and conidial production experiments at constant temperatures of 10 (A), 15 (B), 20 (C), 25 (D), 30 (E) and 35 °C (F); Figure S3: Temperature (°C) and relative humidity (%) recorded at one-hour intervals during the mycelial growth and conidial production experiments held six hours at 15 (A), 20 (B), 25 (C), 30 (D) and 35 °C (E) and then transferred for BOD to 27.4 °C (F) for 10 days; Figure S4: Temperature (°C) and relative humidity (%) recorded at one-hour intervals during the mycelial growth and conidial production experiments held six hours at 15 (A), 20 (B), 25 (C), 30 (D) and 35 °C (E) alternating with 18 h at room temperature (F) for 10 days; Figure S5: Temperature (°C) and relative humidity (%) recorded at one-hour intervals during the virulence experiment held six hours at 15 (A), 20 (B), 30 (C) and 35 °C (D) and then transferred for room temperature (F) for 7 days; Figure S6: Temperature (°C) and relative humidity (%) recorded at one-hour intervals during the virulence experiment held six hours at 15 (A), 20 (B), 25 (C), 30 (D) and 35 °C (E) alternating with 18 h at room temperature (F) for 7 days; Figure S7: Temperature (°C) and relative humidity (%) recorded at one-hour intervals during the virulence experiment at constant temperatures of 15 (A), 20 (B), 25 (C), 30 (D), 35 °C (E) and room temperature (F); Table S1: *p*-values (*p* ≤ value) of the comparisons of confirmed mortality curves for *Bemisia tabaci* nymphs after treatment by *Cordyceps javanica* at different temperatures. Curves were considered significant different at *p* ≤ 0.05.
