# Supplementary material for: Temperature-Dependent Modeling and Spatial Predictions for Identifying Geographical Areas in Brazil Suitable for the Use of Cordyceps javanica in Whitefly Control
Source: J Fungi (Basel). 2025 Feb 8;11(2):125. doi: 10.3390/jof11020125 (PMC11856224; doi:10.3390/jof11020125)
Supplement: Supplementary file 1 [file jof-11-00125-s001.zip › jof-3429715-supplementary.docx]

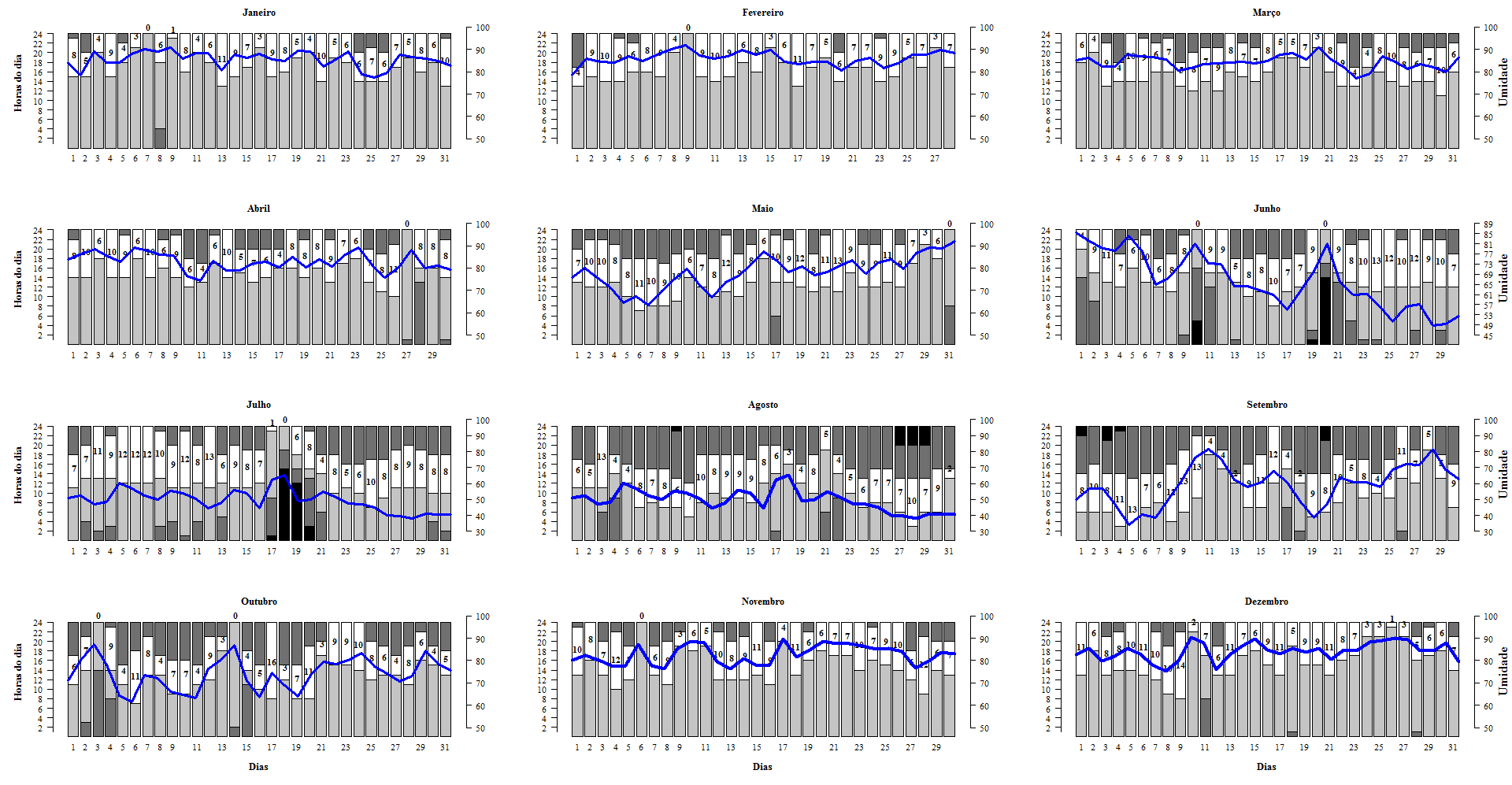

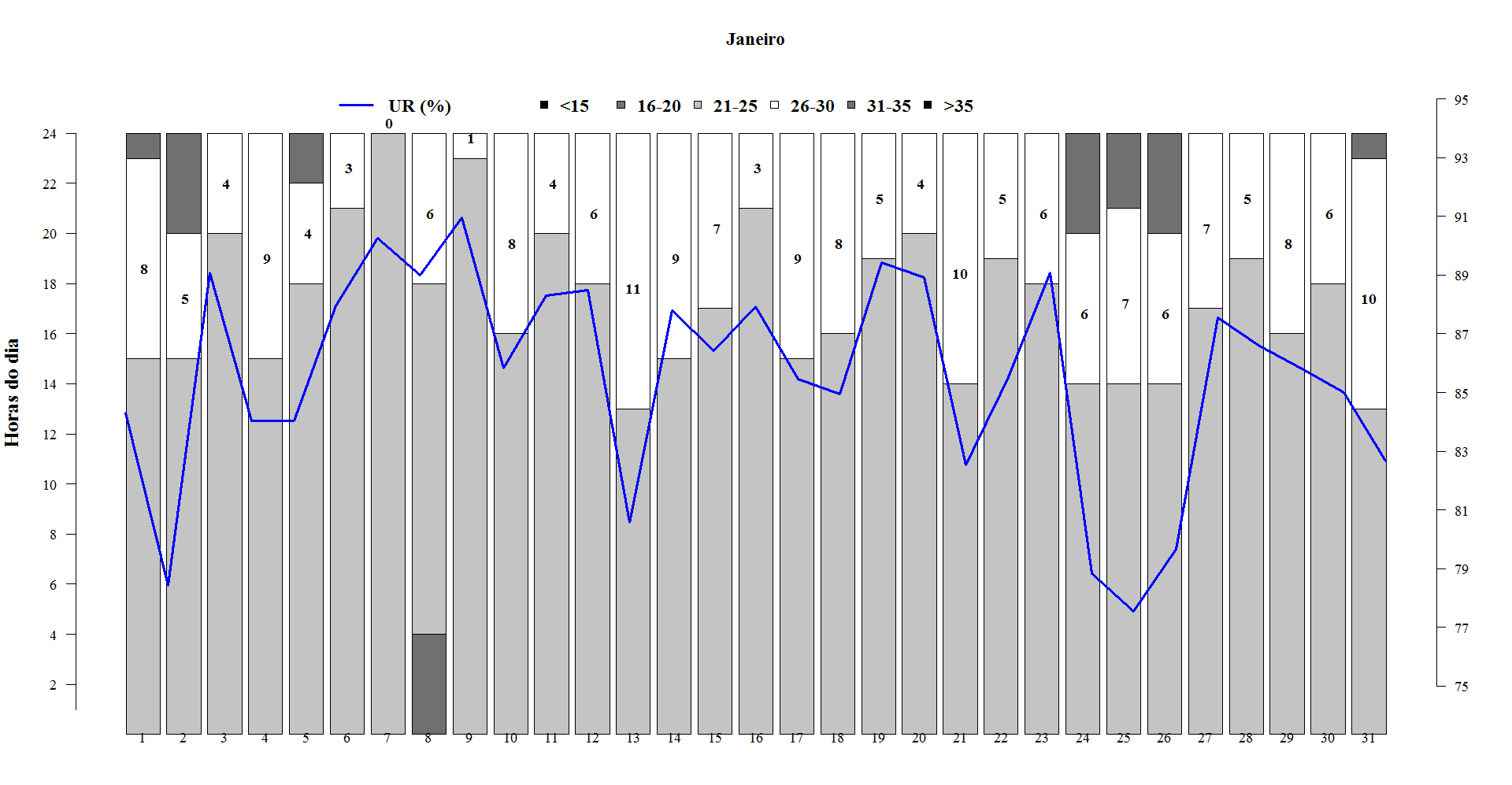


**A**


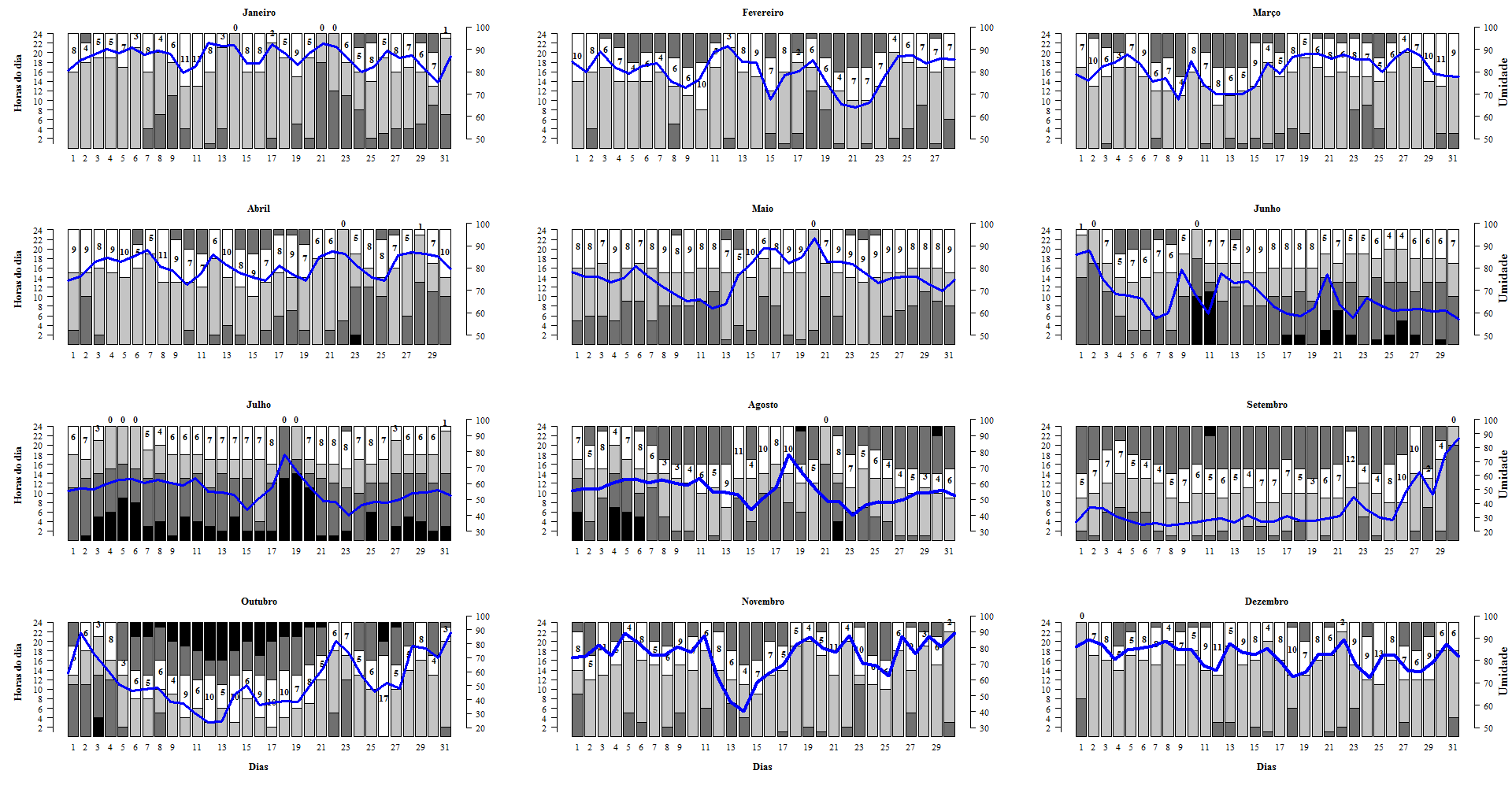


**B**


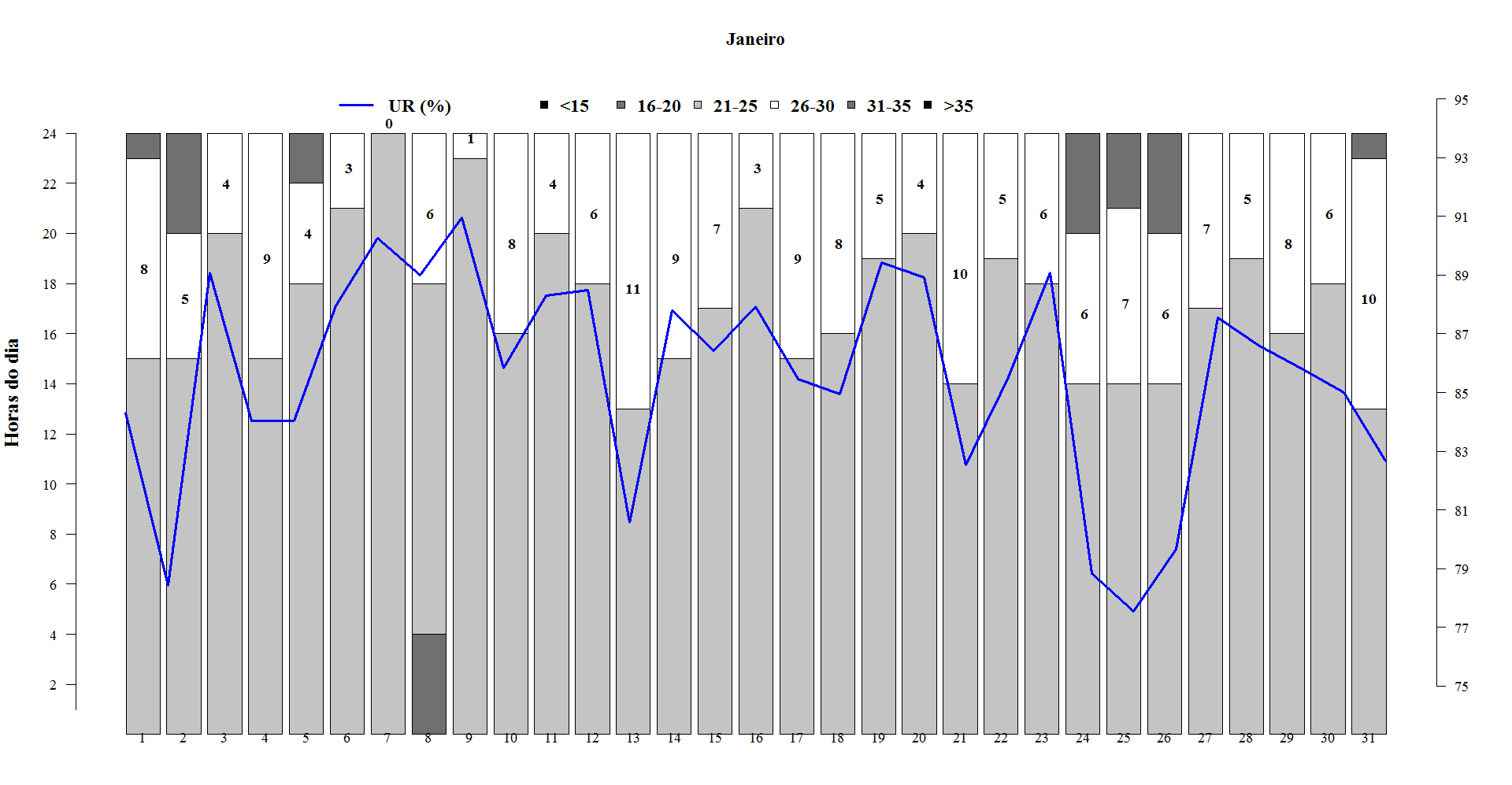


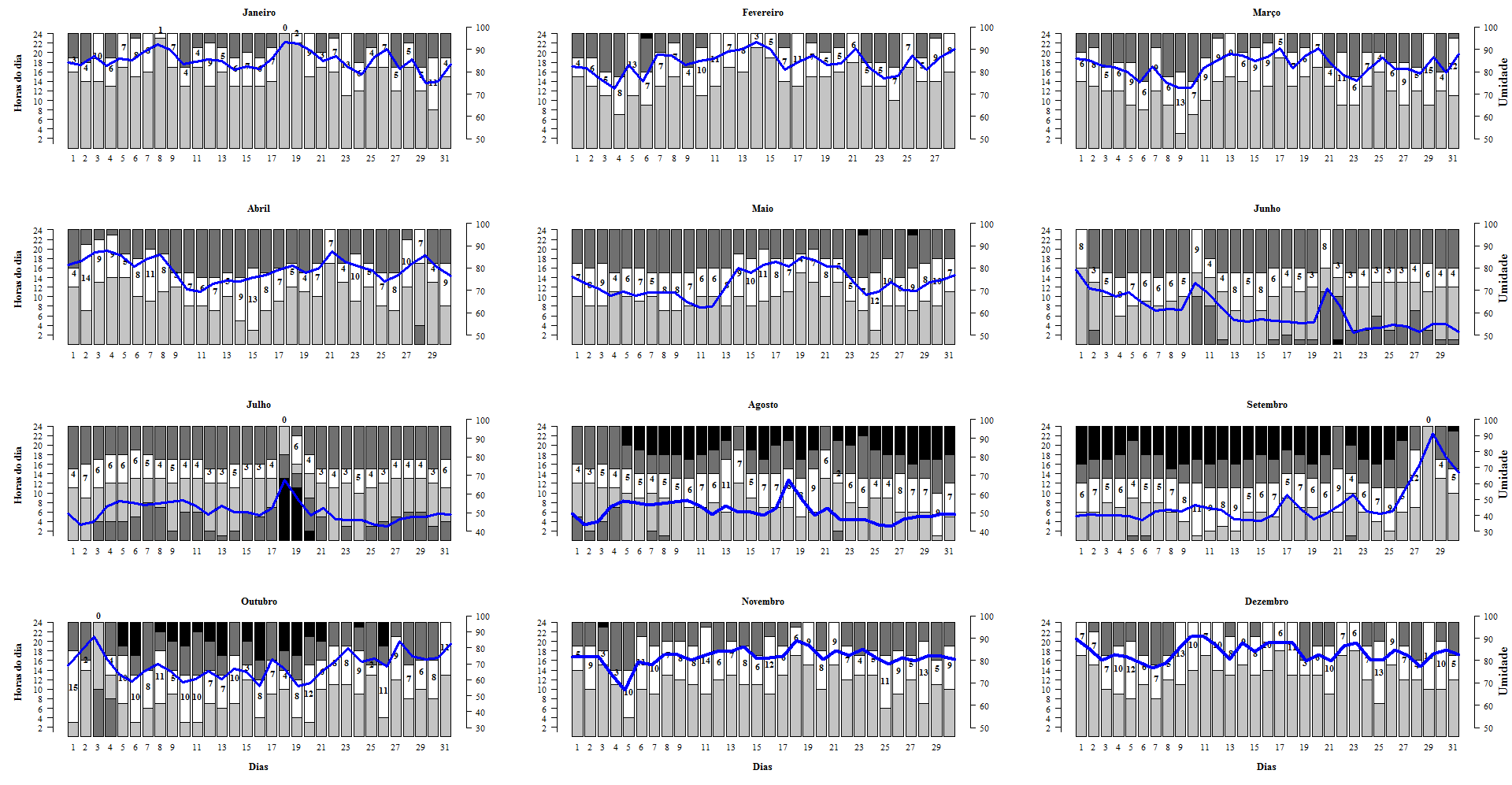


**C**


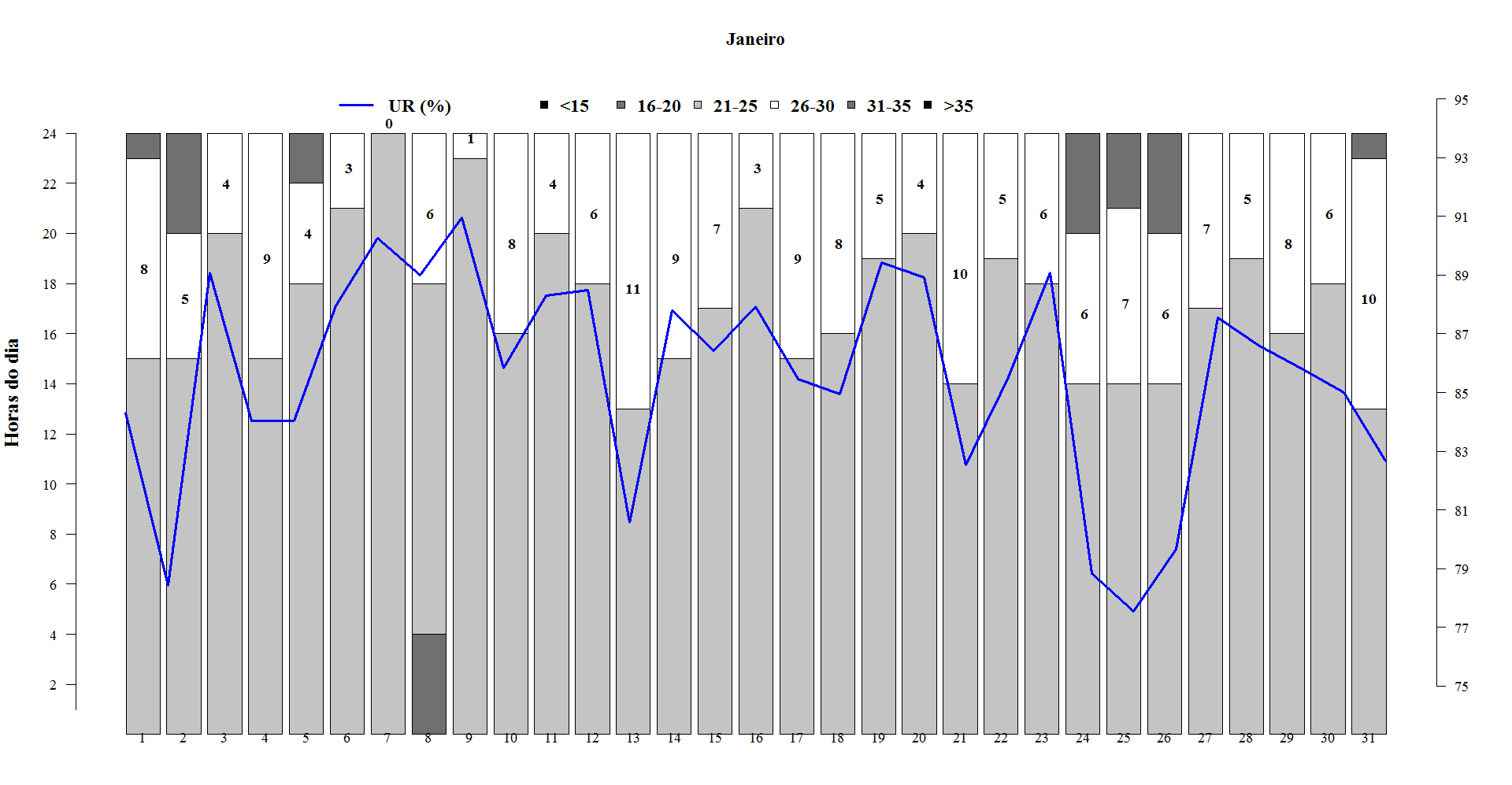


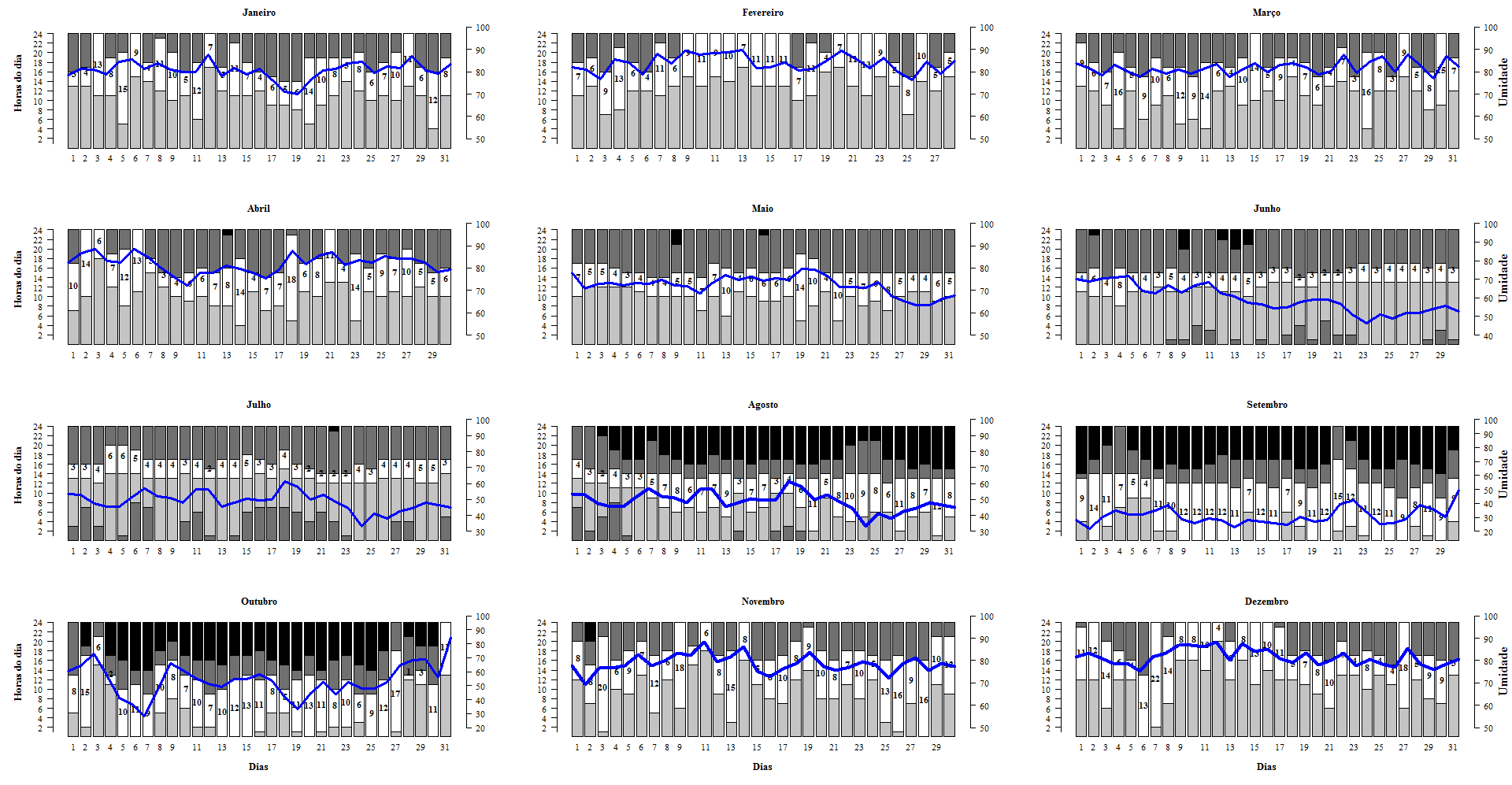


**D**


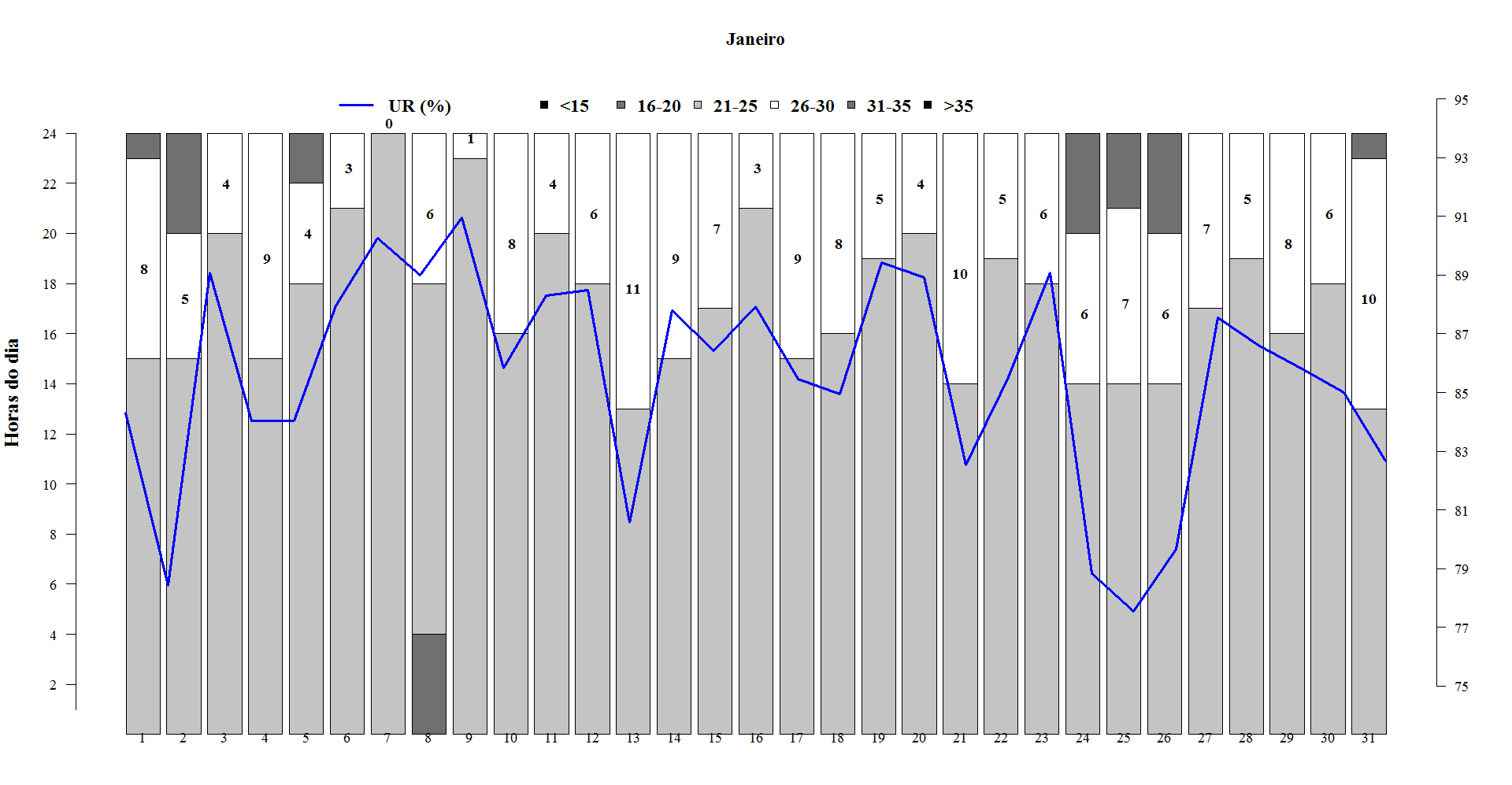


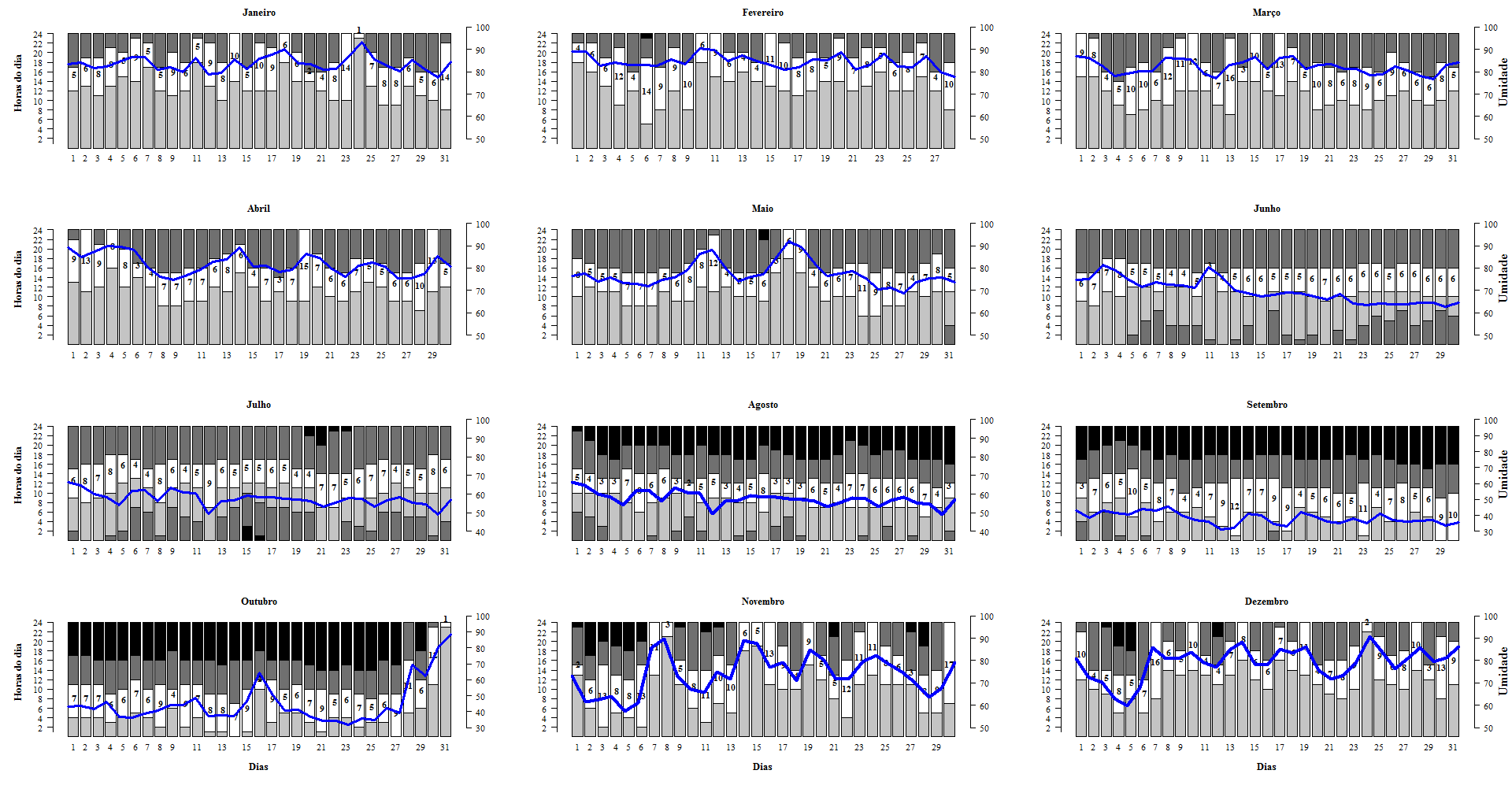


**E**


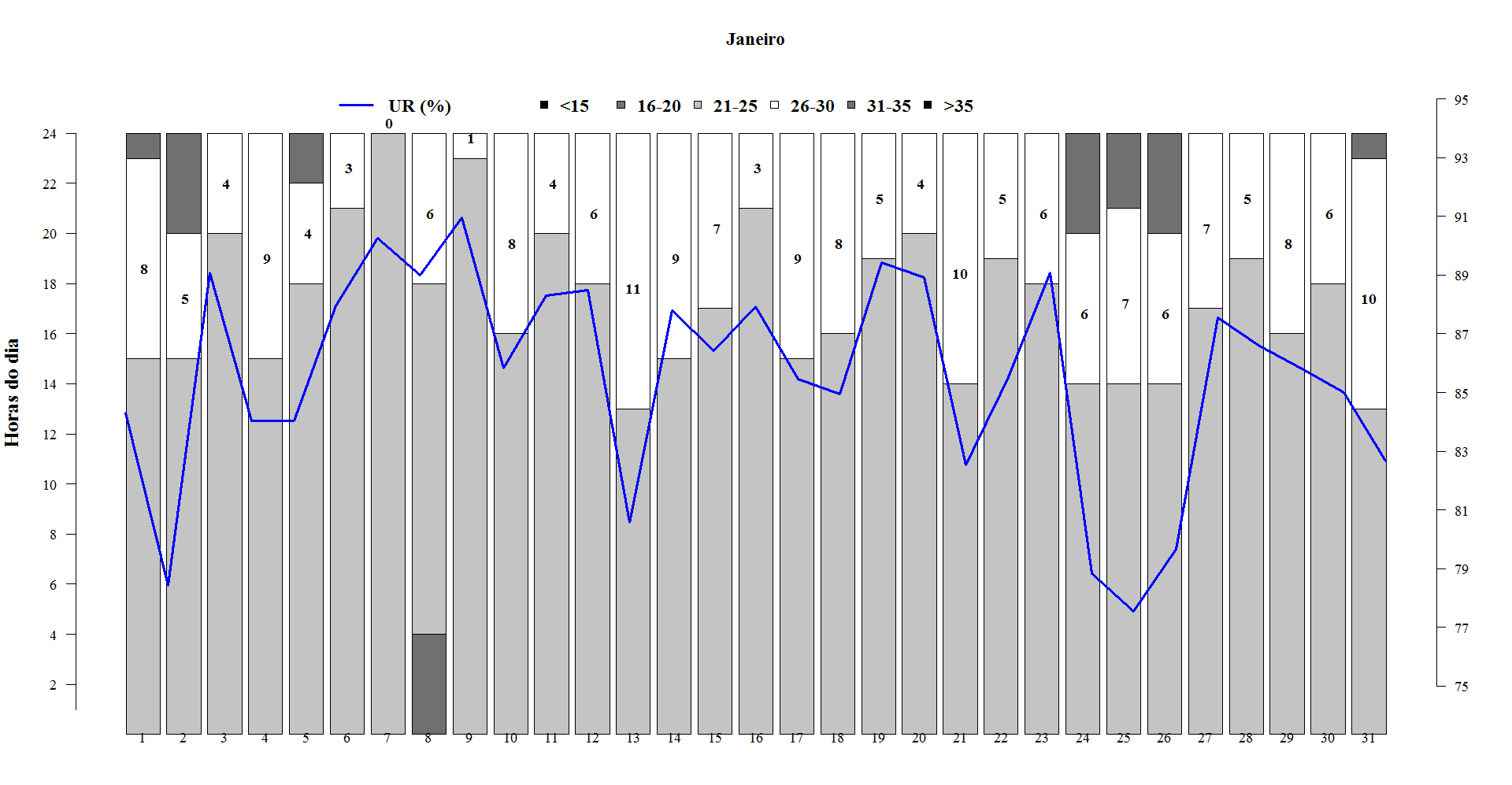


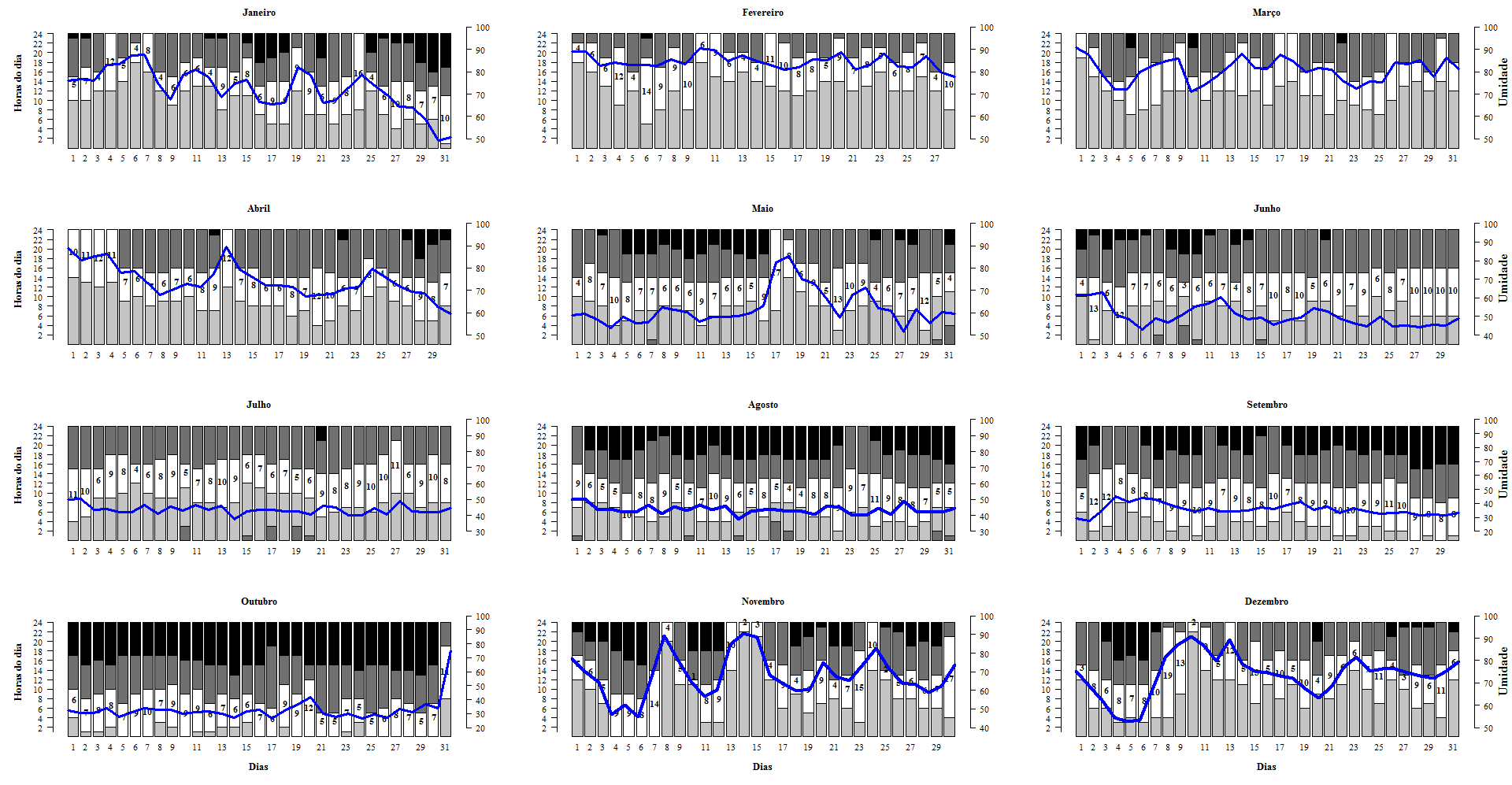


**F**


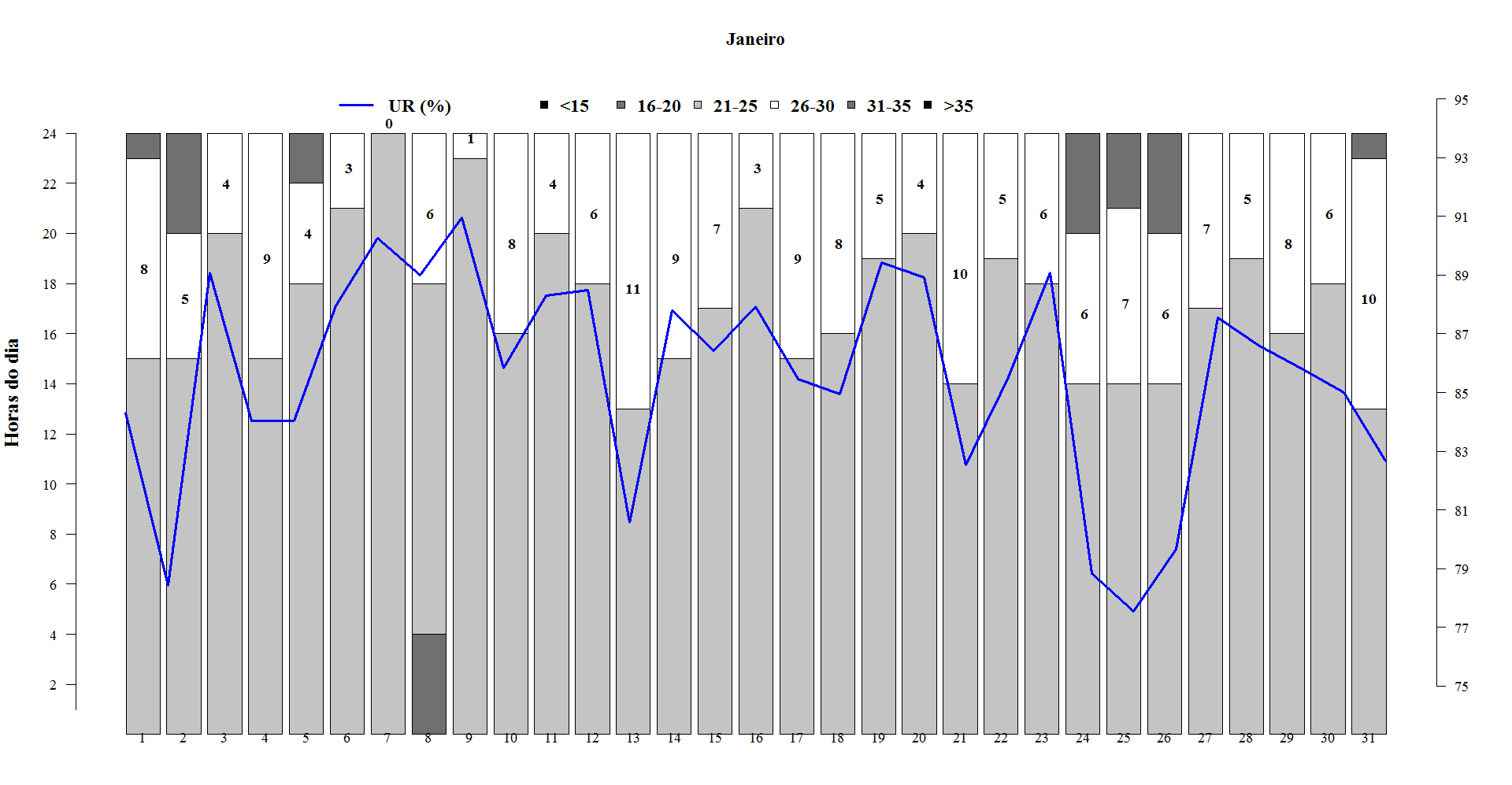


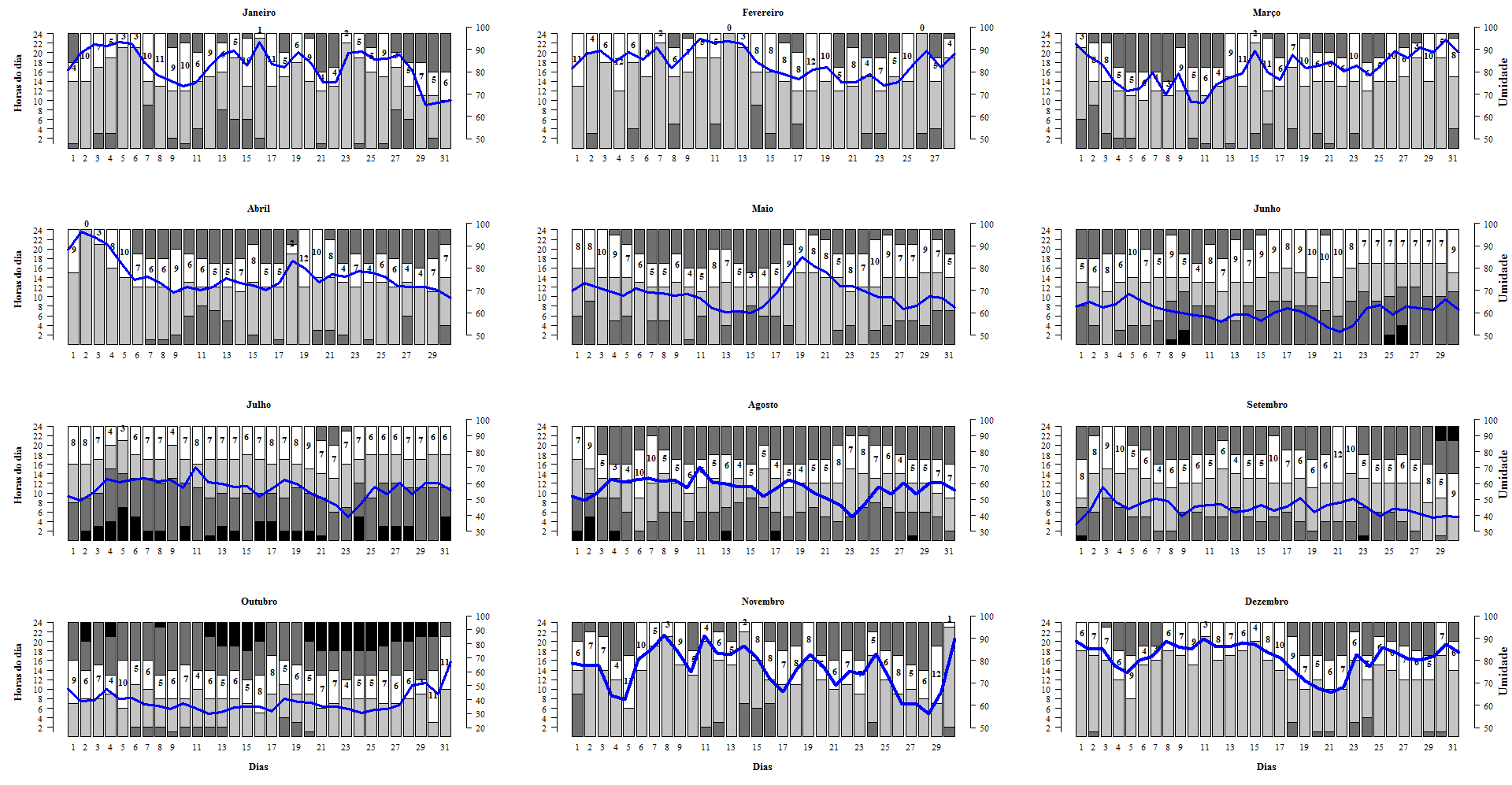


**G**


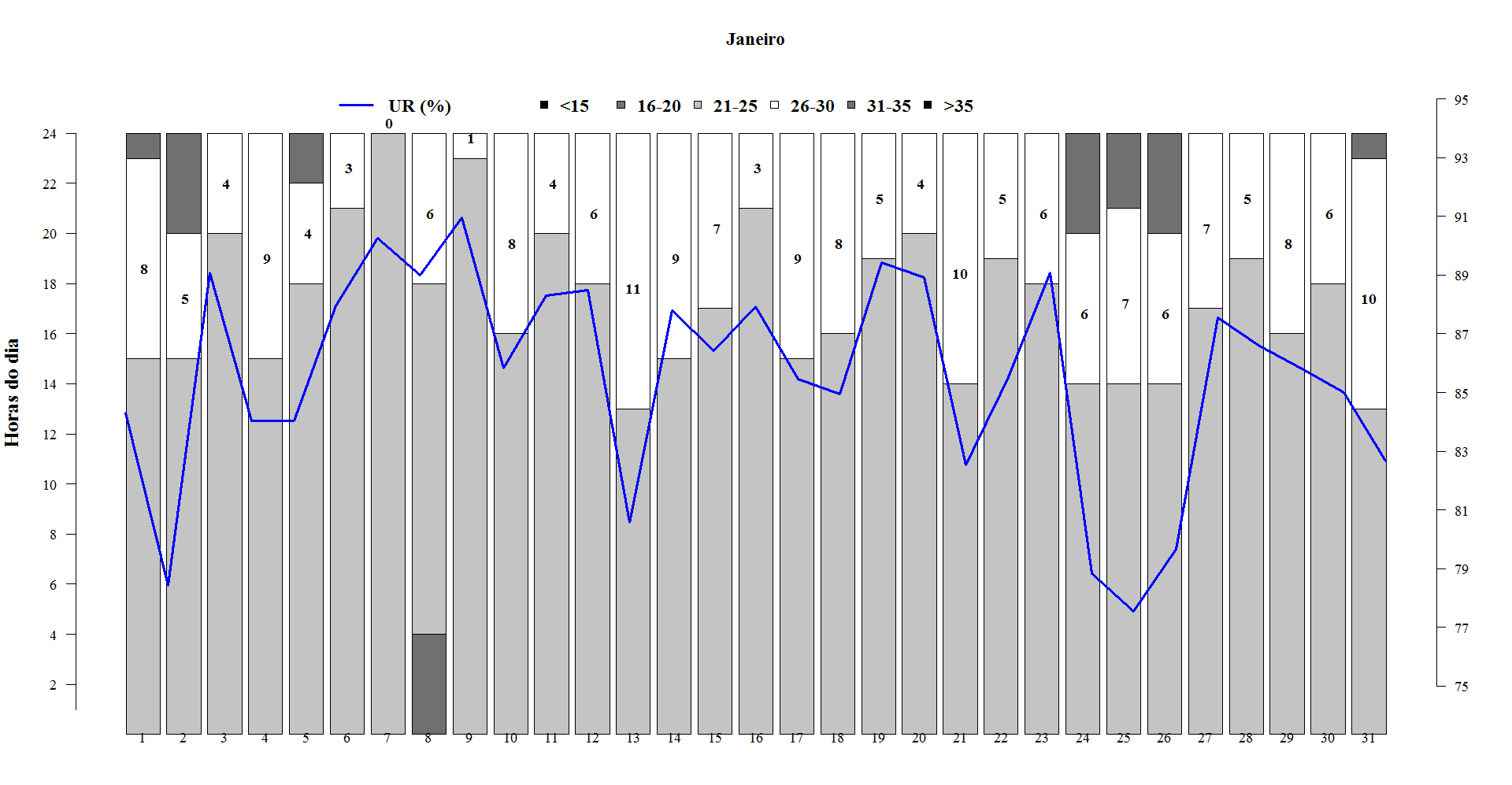


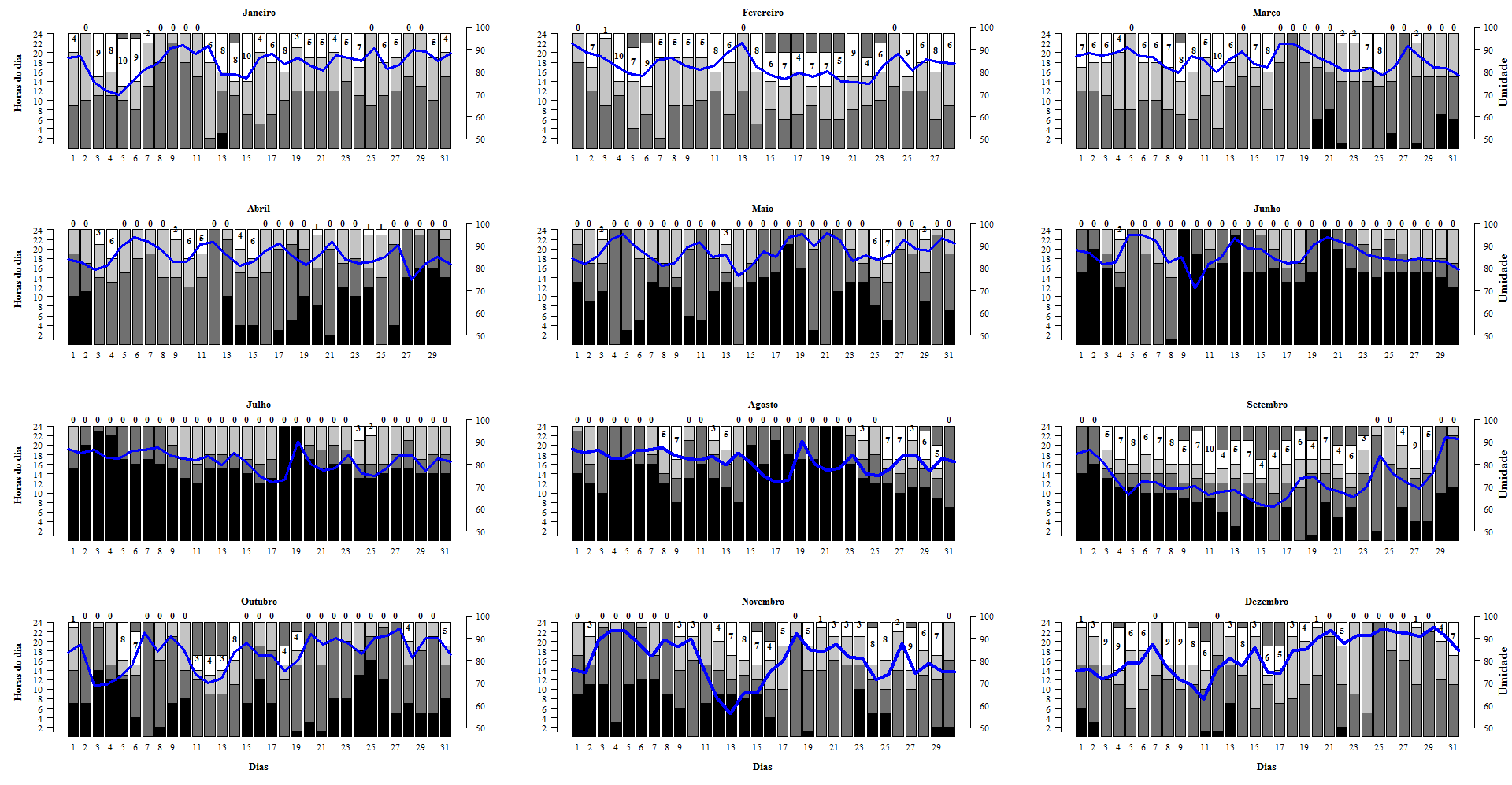


**H**


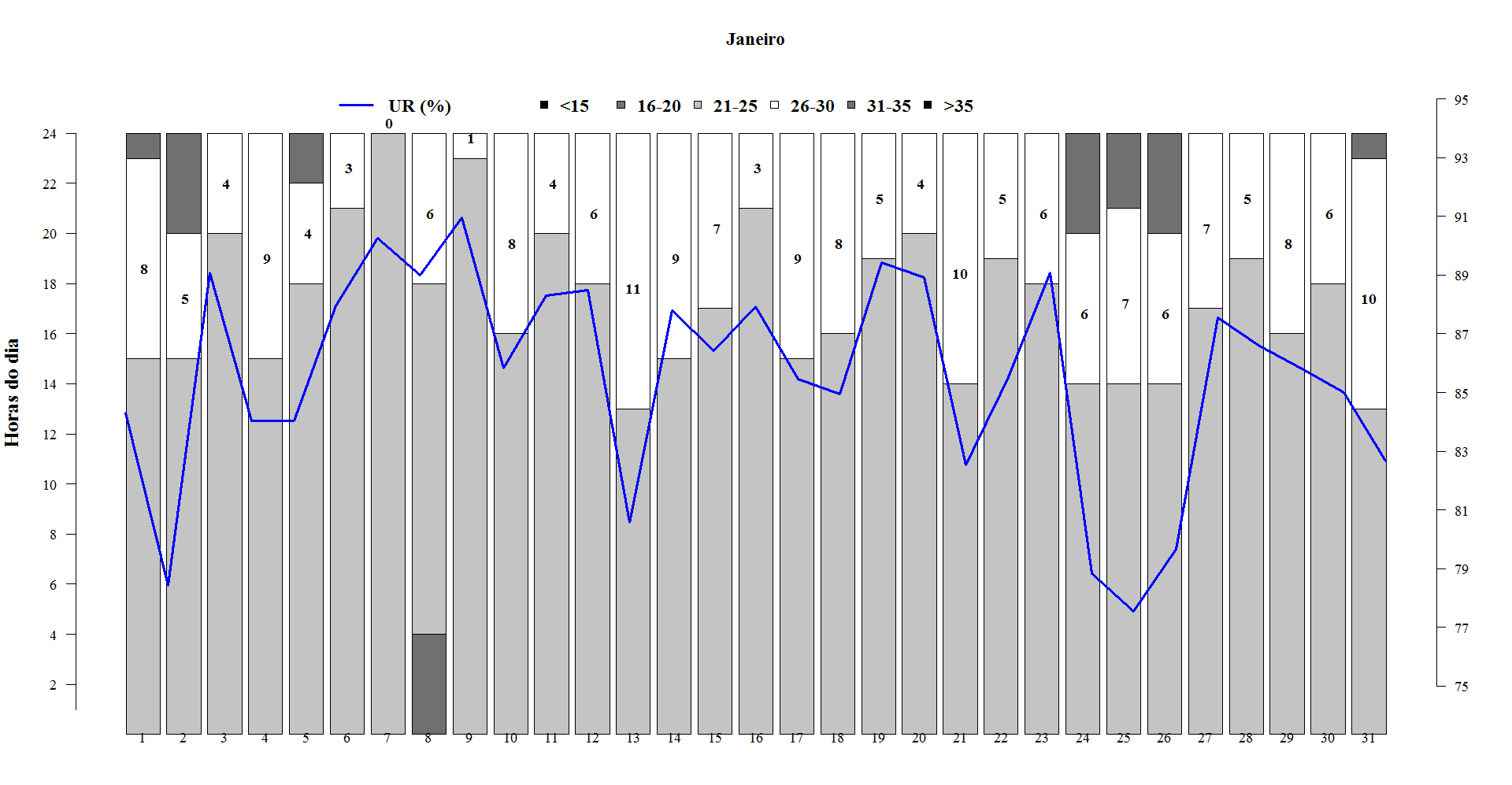


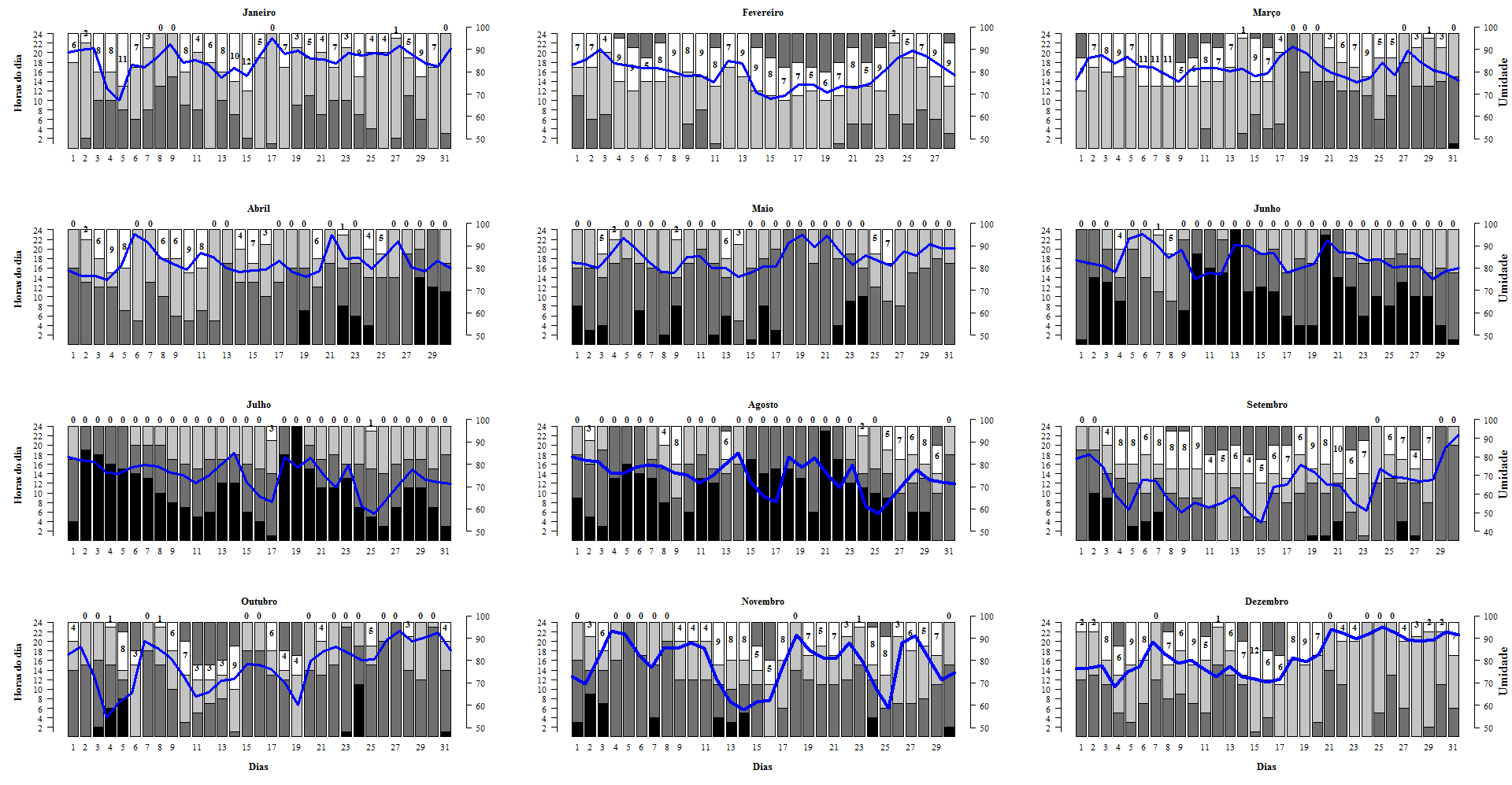


**I**


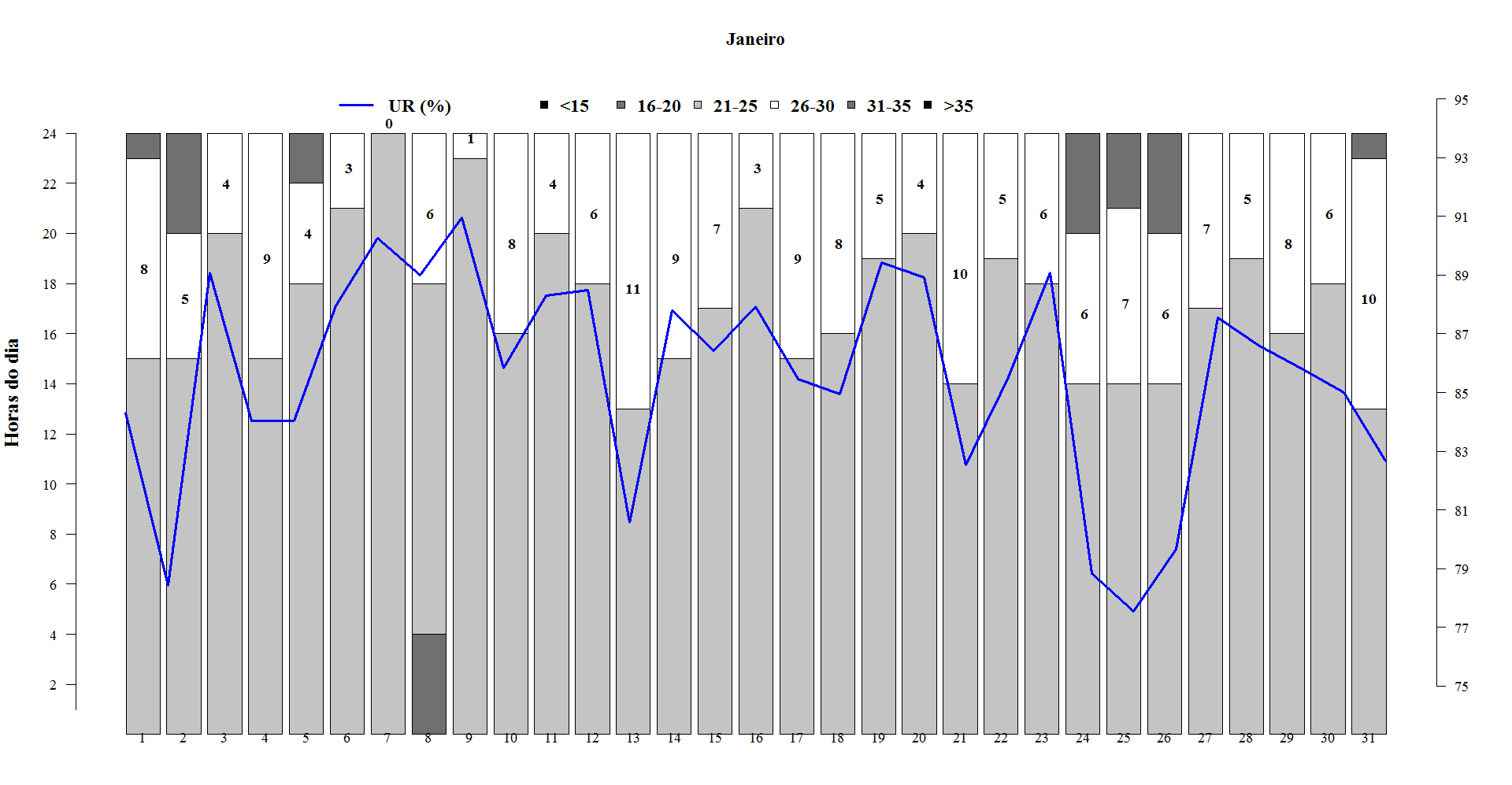


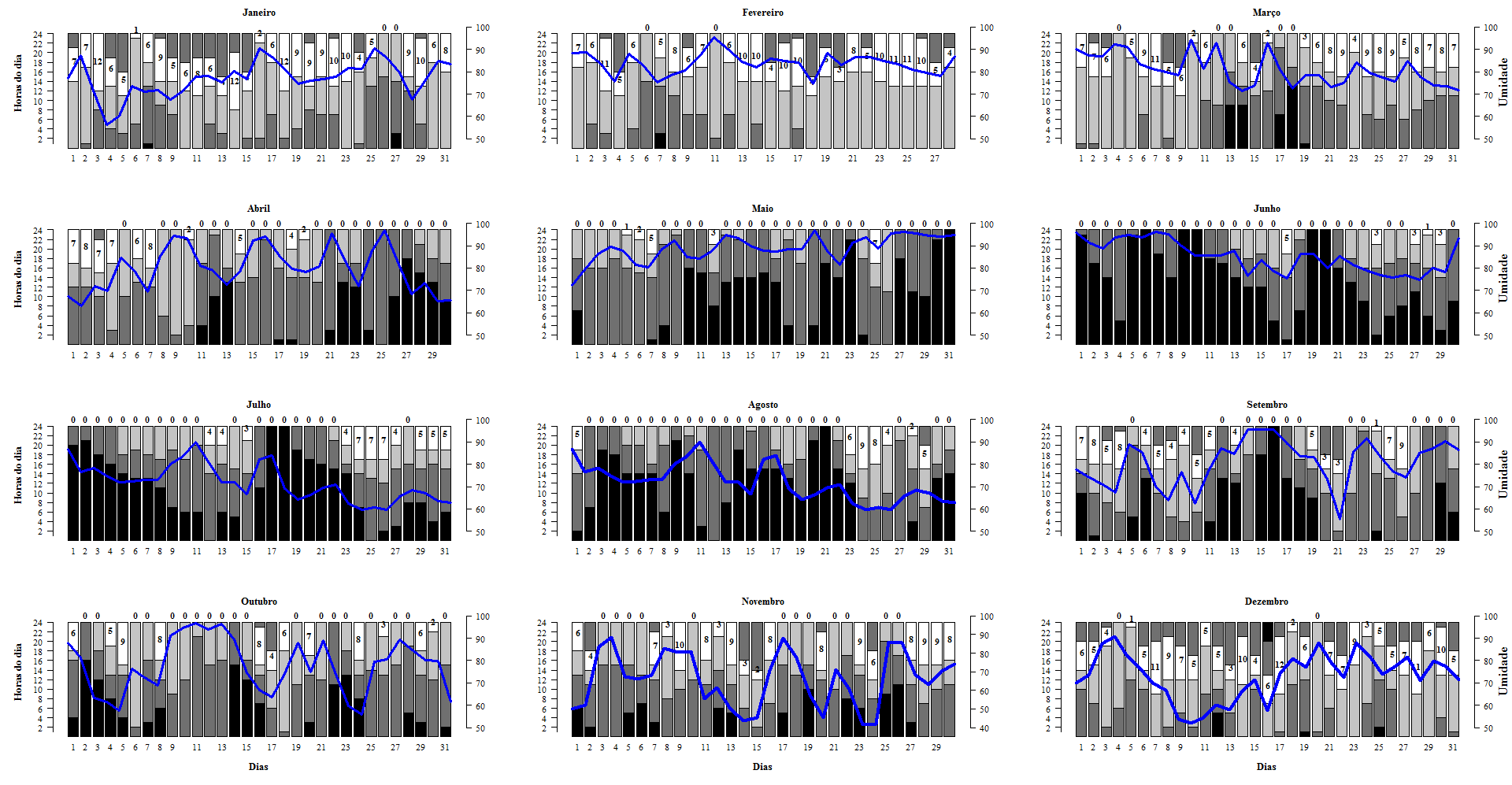


**J**


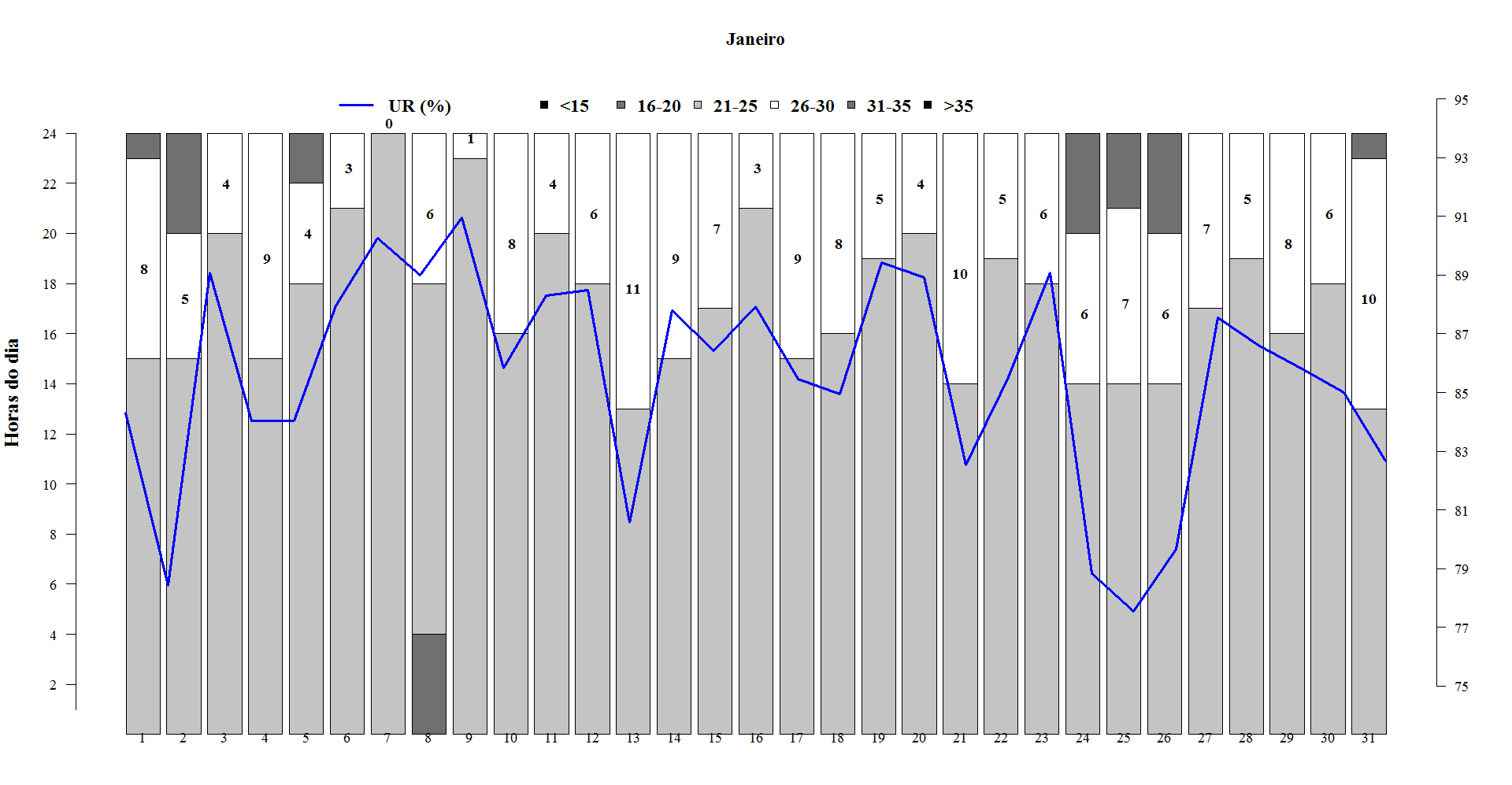


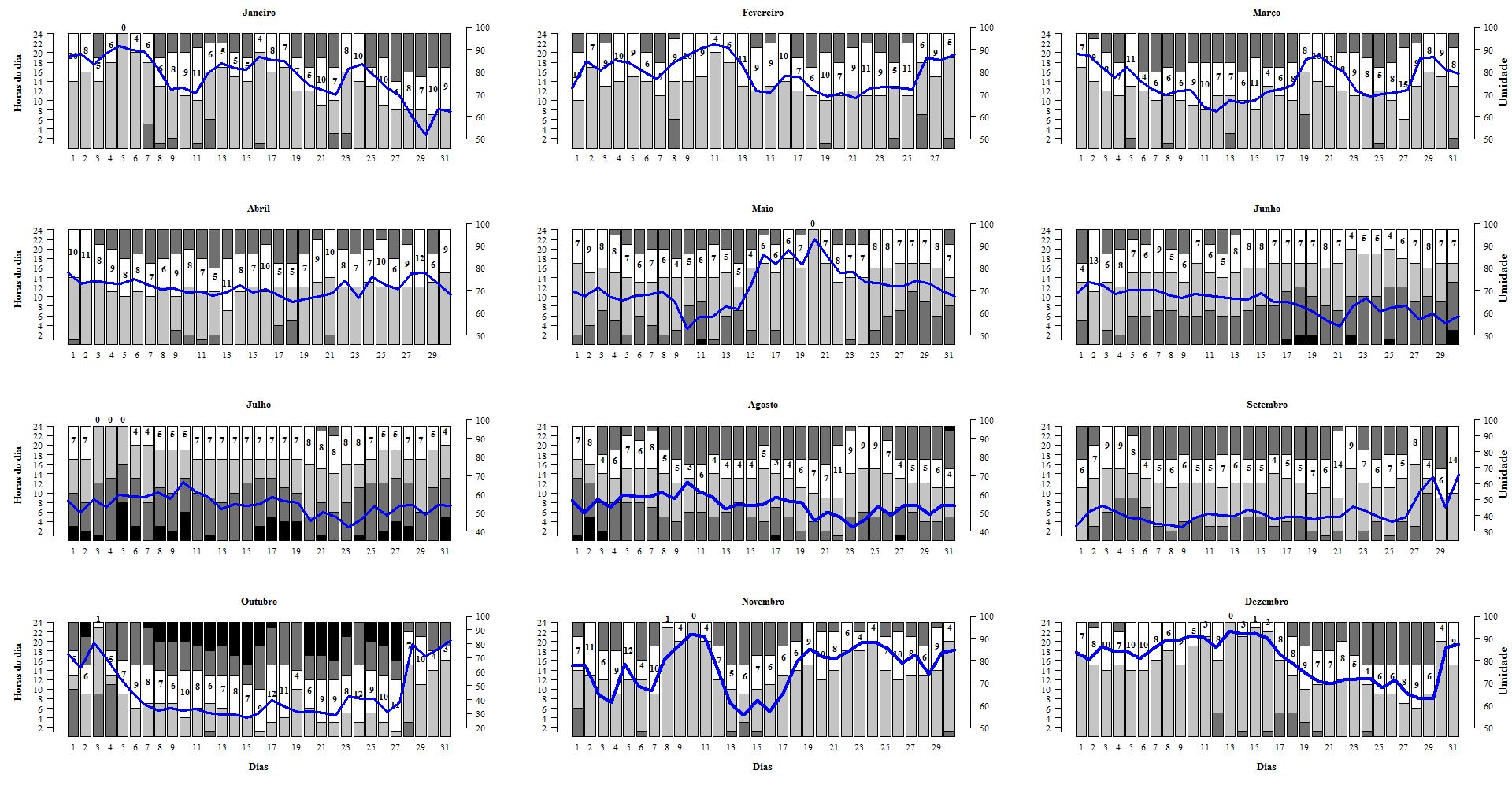


**K**


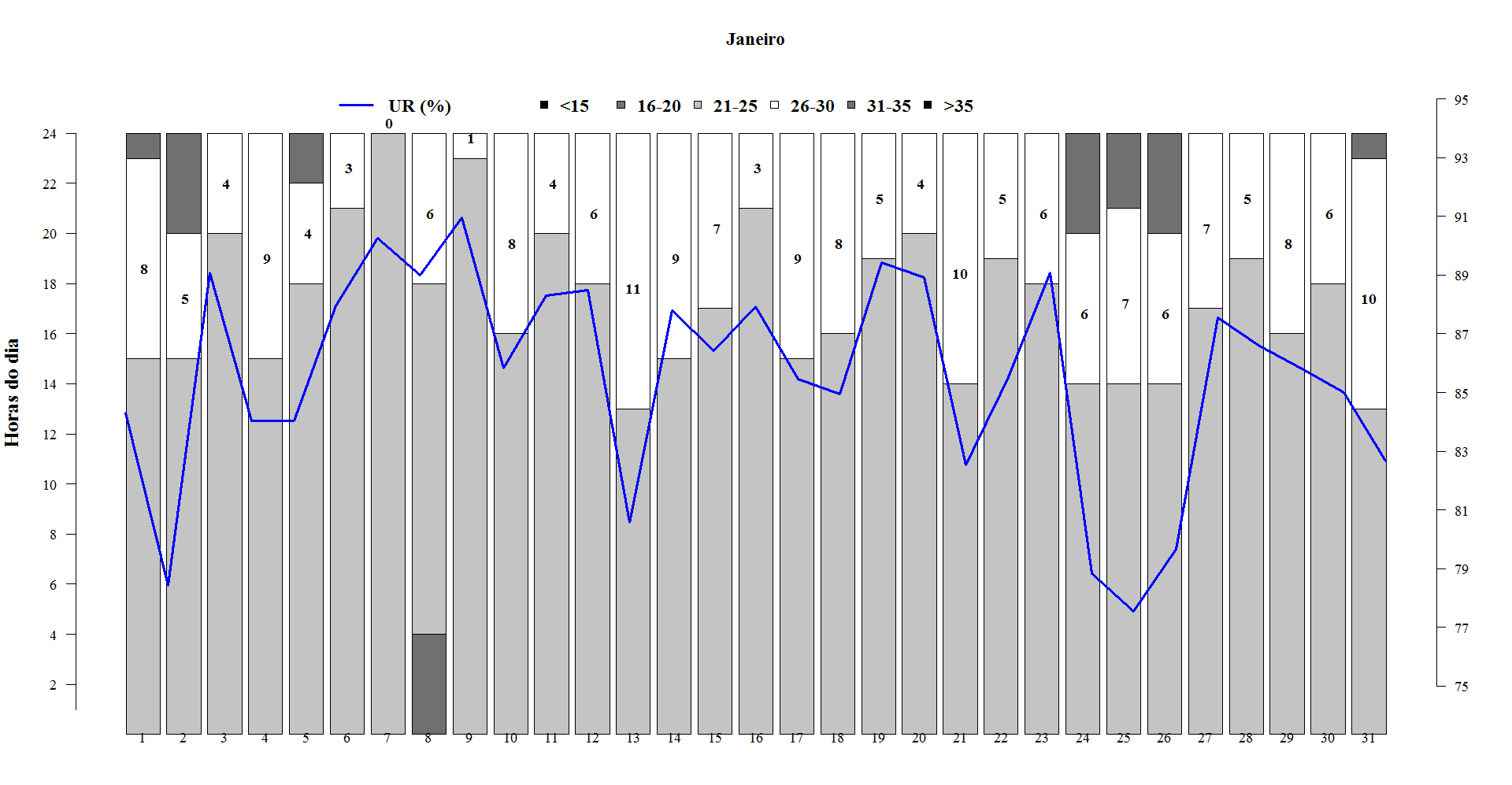


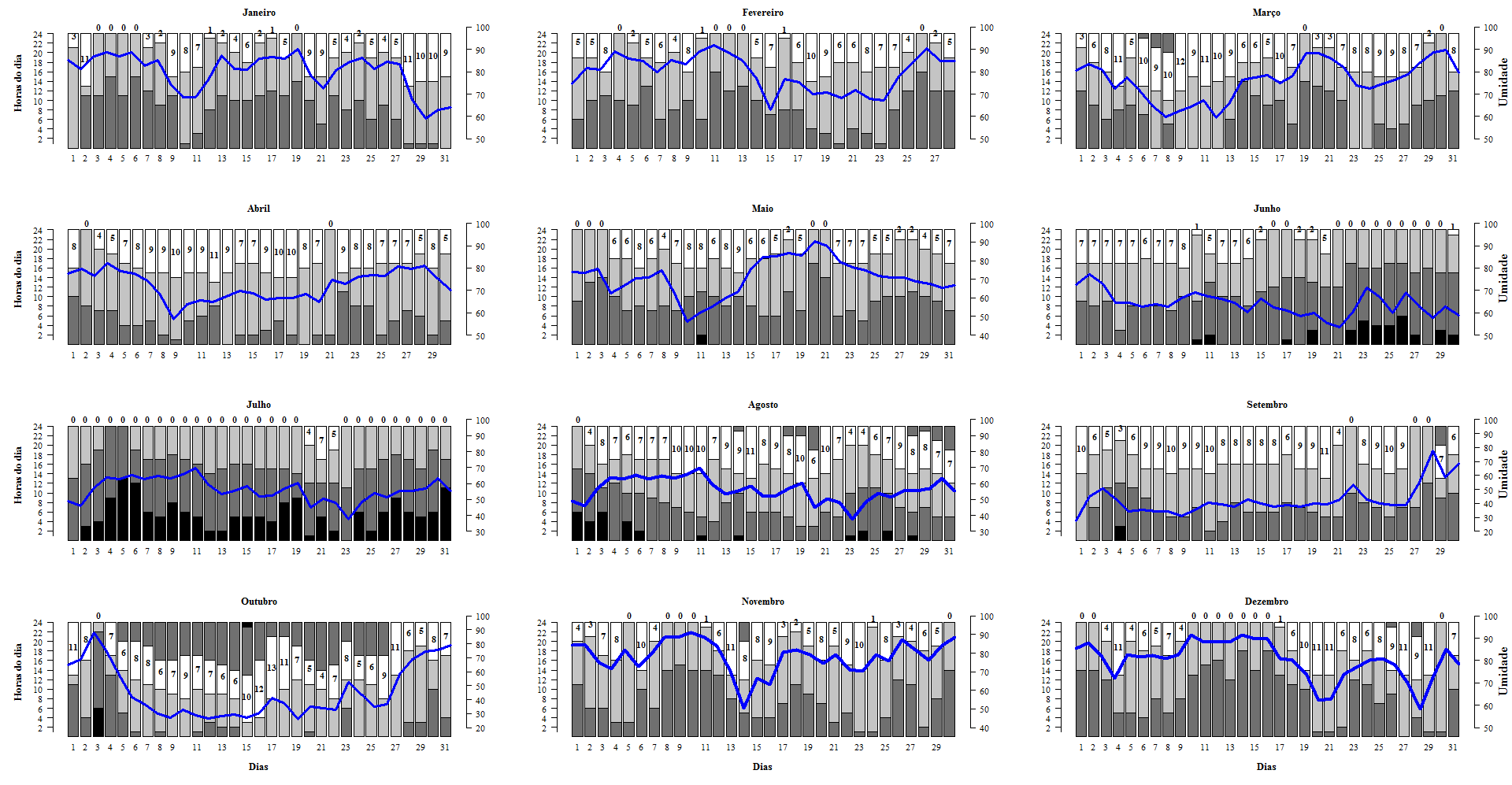


**L**


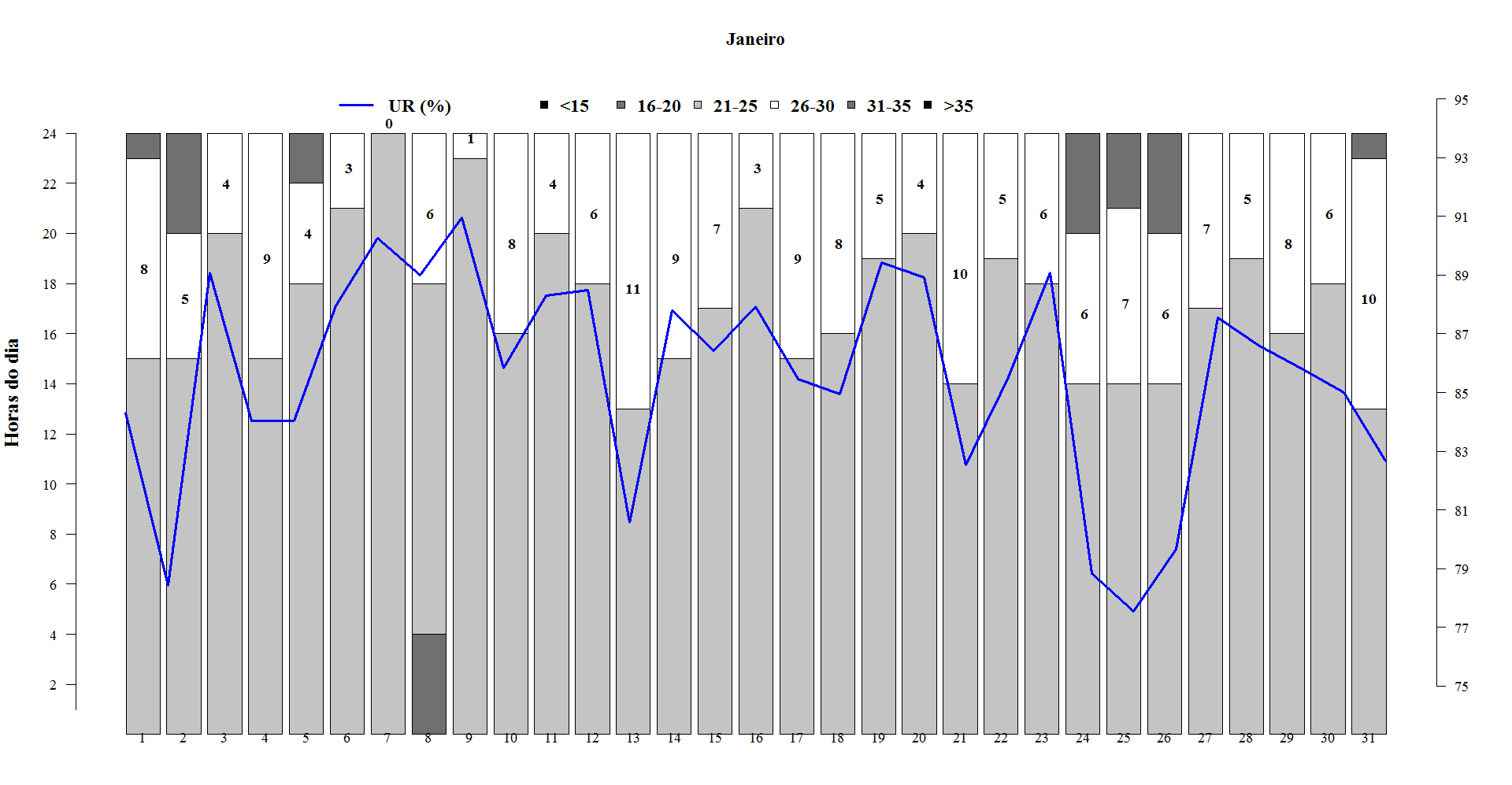


**Figure S1.**  Number of hours (represented by bars) that temperatures remain within the ranges of <15 °C, 16-20 °C, 21-25 °C, 26-30 °C, 31-35 °C, and >35 °C, and relative humidity (illustrated by a blue line), for the months of January through December 2017 in Brazil for the city and state of Vilhena, Rondônia (A); Rio Verde, Goiás (B); Sorriso, Mato Grosso (C); Formoso do Araguaia, Tocantins (D); Balsas, Maranhão (E); Bom Jesus, Piauí (F); Luis Eduardo Magalhães, Bahia (G); Castro, Paraná (H); Itapeva, São Paulo (I); Tupanciretã, Rio Grande do Sul (J); Unaí, Minas Gerais (K); and Brasília, Distrito Federal (L).


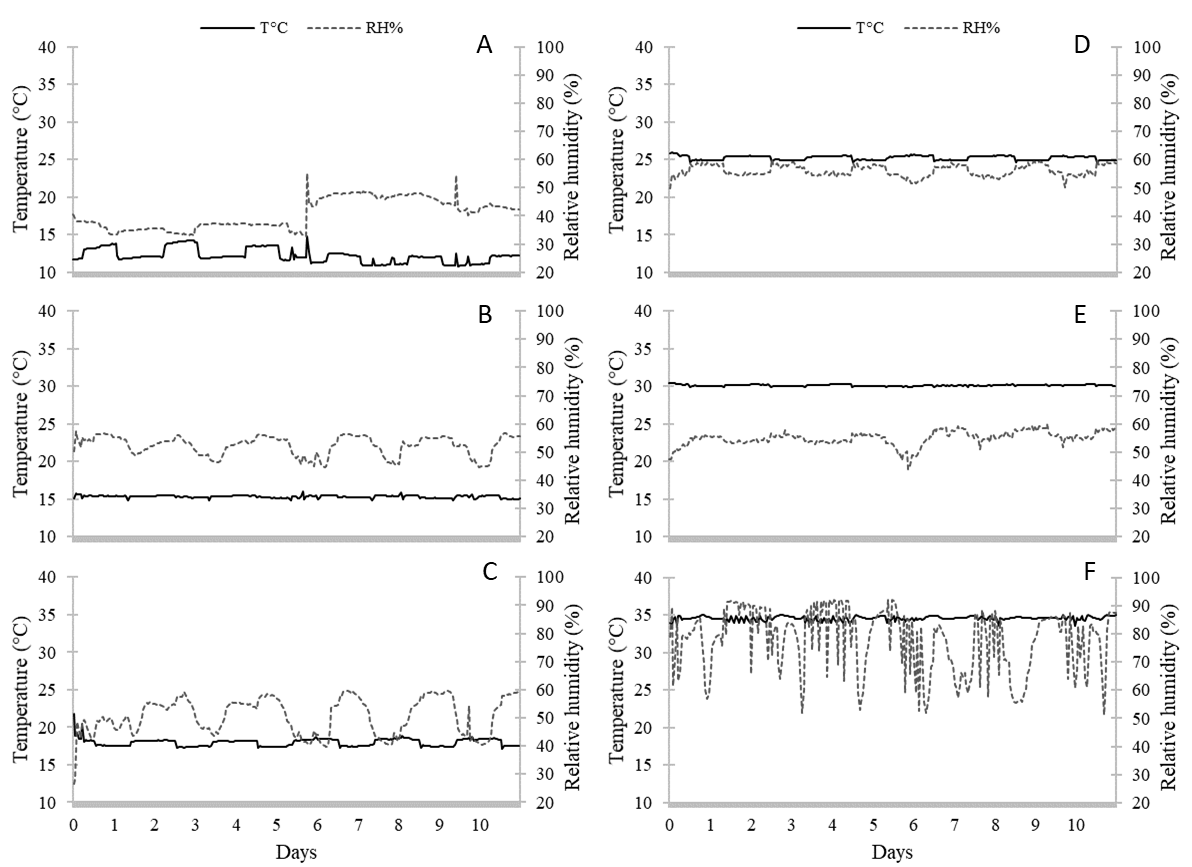


**Figure S2.** Temperature (°C) and relative humidity (%) recorded at one-hour intervals during the mycelial growth and conidial production experiments at constant temperatures of 10 (A), 15 (B), 20 (C), 25 (D), 30 (E) and 35 °C (F).


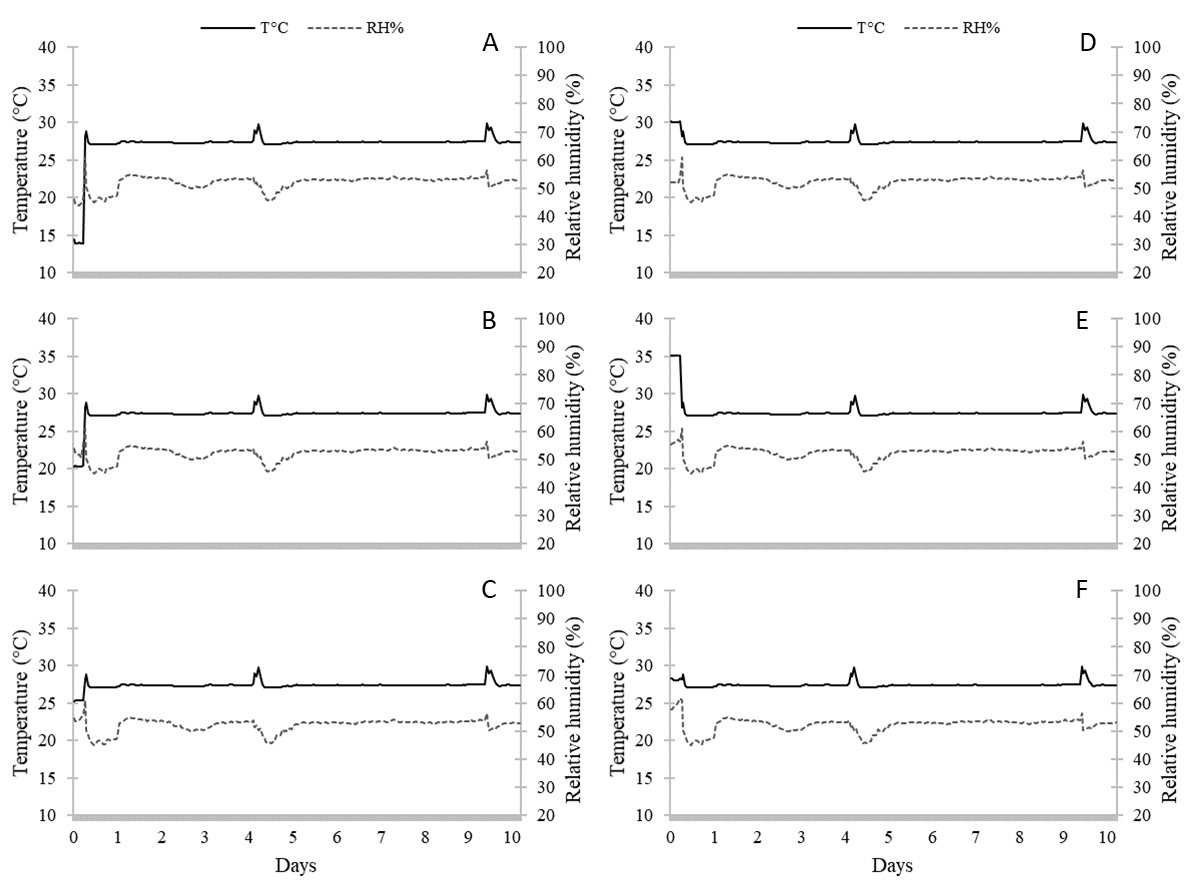


**Figure S3.** Temperature (°C) and relative humidity (%) recorded at one-hour intervals during the mycelial growth and conidial production experiments held six hours at 15 (A), 20 (B), 25 (C), 30 (D) and 35 °C (E) and then transferred for BOD to 27.4 °C (F) for 10 days.

**
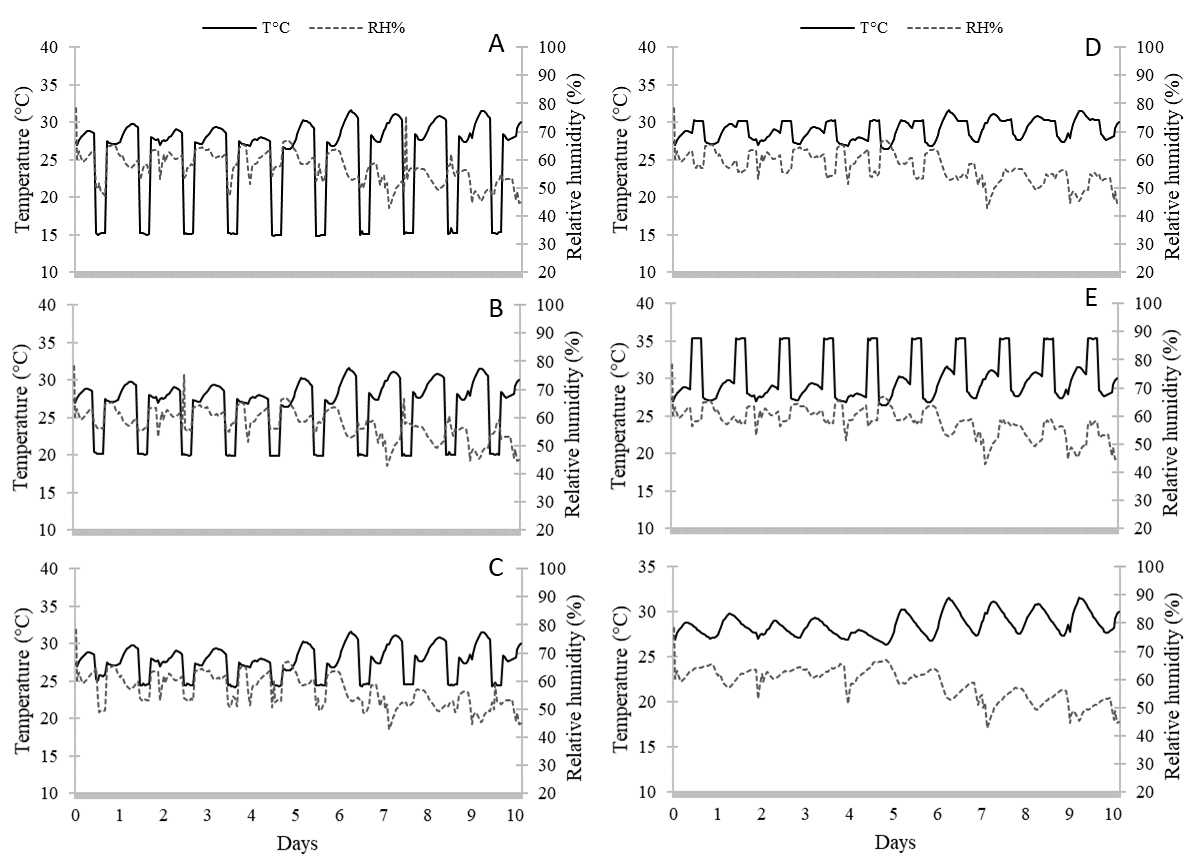
**

**Figure S4.** Temperature (°C) and relative humidity (%) recorded at one-hour intervals during the mycelial growth and conidial production experiments held six hours at 15 (A), 20 (B), 25 (C), 30 (D) and 35 °C (E) alternating with 18 h at room temperature (F) for 10 days.


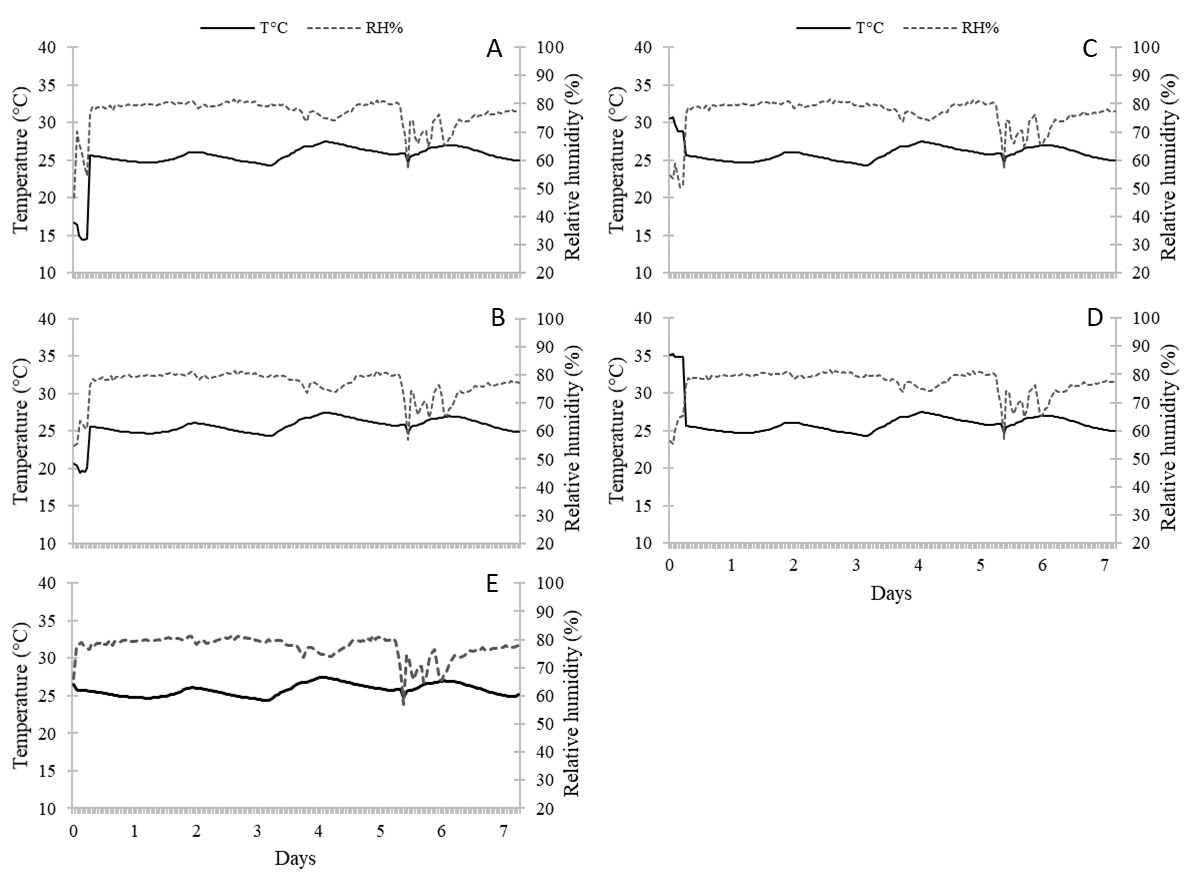


**Figure S5.** Temperature (°C) and relative humidity (%) recorded at one-hour intervals during the virulence experiment held six hours at 15 (A), 20 (B), 30 (C) and 35 °C (D) and then transferred for room temperature (F) for 7 days.

**
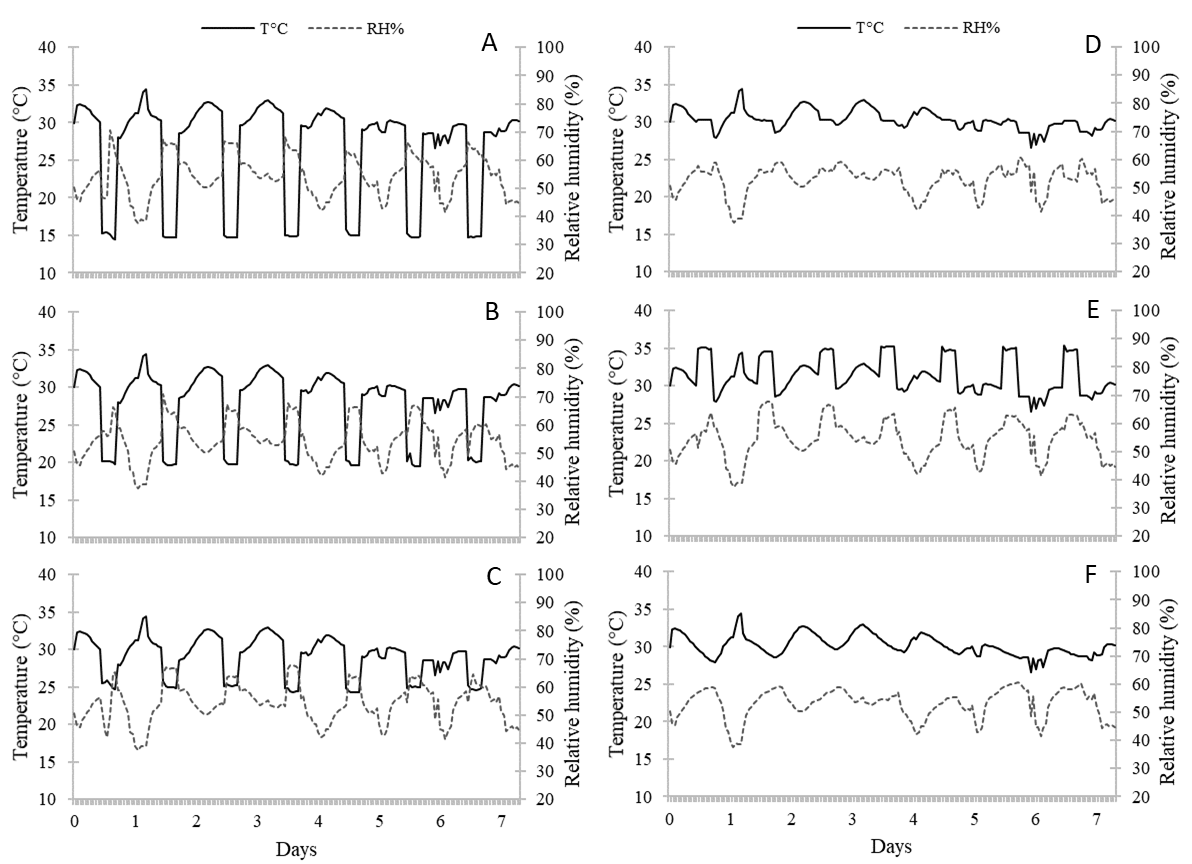
**

**Figure S6.** Temperature (°C) and relative humidity (%) recorded at one-hour intervals during the virulence experiment held six hours at 15 (A), 20 (B), 25 (C), 30 (D) and 35 °C (E) alternating with 18 h at room temperature (F) for 7 days.


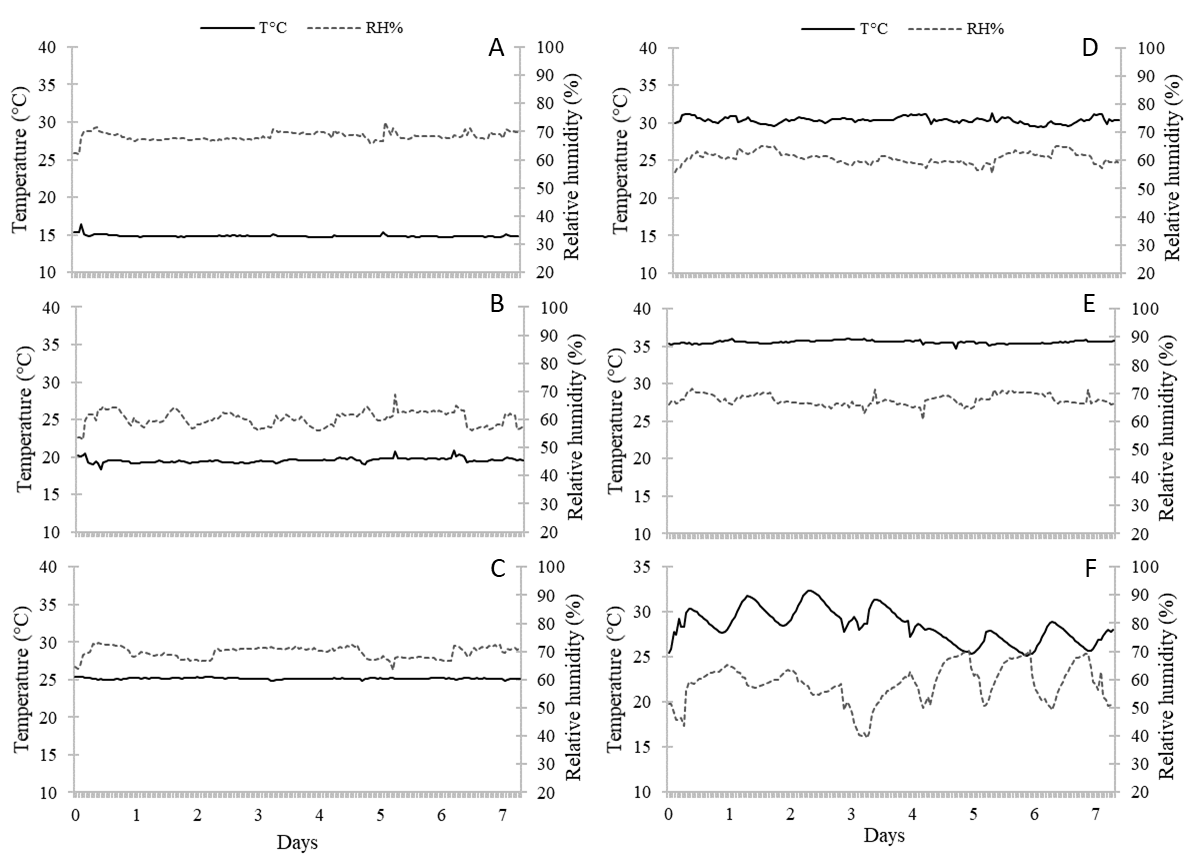


**Figure S7.** Temperature (°C) and relative humidity (%) recorded at one-hour intervals during the virulence experiment at constant temperatures of 15 (A), 20 (B), 25 (C), 30 (D), 35 °C (E) and room temperature (F).

**Table S1.** *p*-values (*p* ≤ value) of the comparisons of confirmed mortality curves for *Bemisia tabaci* nymphs after treatment by *Cordyceps javanica* at different temperatures. Curves were considered significant different at *p* ≤ 0.05.

| **CONFIRMED MORTALITY** | | | | | | | | |
| --- | --- | --- | --- | --- | --- | --- | --- | --- |
| Experiment 1 | | | | | | | | |
| Temperature (°C) | | 15 | | 20 | | | 30 | |
| 20 | | 0.578 | | . | | | . | |
| 30 | | 0.512 | | 0.608 | | | . | |
| 35 | | 0.439 | | 0.610 | | | 0.812 | |
| Experiment 2 | | | | | | | | |
| Temperature (°C) | 15 | | 20 | | 25 | 30 | | 35 |
| 20 | 0.625 | | . | | . | . | | . |
| 25 | 0.254 | | 0.448 | | . | . | | . |
| 30 | 0.269 | | 0.476 | | 0.867 | . | | . |
| 35 | 0.782 | | 0.579 | | 0.242 | 0.248 | | . |
| 27.6 | 0.535 | | 0.819 | | 0.479 | 0.520 | | 0.532 |
| Experiment 3 | | | | | | | | |
| Temperature (°C) | 15 | | 20 | | 25 | 30 | | 35 |
| 20 | 0.799 | | . | | . | . | | . |
| 25 | 0.003 | | 0.007 | | . | . | | . |
| 30 | <0.001 | | <0.001 | | 0.199 | . | | . |
| 35 | 0.784 | | 0.699 | | 0.004 | <0.001 | | . |
| 28.4 | 0.210 | | 0.299 | | 0.094 | 0.004 | | 0.208 |
